# Bloodstream Infections in Hospitalized Patients with COVID-19: A Systematic Review and Meta-Analysis

**DOI:** 10.3390/microorganisms9102016

**Published:** 2021-09-23

**Authors:** Mariachiara Ippolito, Barbara Simone, Carlotta Filisina, Francesca Romana Catalanotto, Giulia Catalisano, Claudia Marino, Giovanni Misseri, Antonino Giarratano, Andrea Cortegiani

**Affiliations:** 1 Department of Surgical, Oncological and Oral Science (Di.Chir.On.S.), University of Palermo, 90127 Palermo, Italy; mariachiara.ippolito@community.unipa.it (M.I.); barbara.simone1993@gmail.com (B.S.); carlottafilisina@gmail.com (C.F.); francesca.catalanott@libero.it (F.R.C.); giuliacatalisano@gmail.com (G.C.); dott.ssacmarino@gmail.com (C.M.); antonino.giarratano@unipa.it (A.G.); 2 Fondazione Giglio, 90015 Cefalù, Italy; giovannimisseri1987@gmail.com; 3 Department of Anaesthesia, Intensive Care and Emergency, Policlinico Paolo Giaccone, 90127 Palermo, Italy

**Keywords:** bloodstream infections, COVID-19, review

## Abstract

Background: Little is known about the occurrence of bloodstream infections in hospitalized patients with COVID-19 and the related clinical consequences. The aim of this systematic review and meta-analysis was to estimate the pooled occurrence of BSIs among hospitalized patients with COVID-19 and mortality of this patient population. Methods: A systematic search was performed on PubMed, EMBASE, and Web of Science from inception to 19 April 2021. The primary outcome was the occurrence of BSIs among hospitalized patients with COVID-19. The secondary outcome was mortality at the longest available follow-up. Results: Forty-six studies met the inclusion criteria, with a total of 42,694 patients evaluated. The estimated occurrence of BSIs was 7.3% (95% CI 4.7–1.1%) among hospitalized patients with COVID-19, with a mortality rate of 41% (95% CI 30%–52.8%). The subgroup analysis conducted on patients admitted to ICU provided an estimated occurrence of 29.6% (95% CI 21.7%–38.8%). A higher occurrence of BSI was observed in patients with COVID-19, in comparison with patients without COVID-19 (OR 2.77; 95% CI 1.53–5.02; *p* < 0.001). Conclusions: Our analysis estimated the occurrence of BSIs among hospitalized patients with COVID-19 at around 7%. A four-times higher occurrence was estimated among patients admitted to ICU.

## 1. Introduction

Bloodstream infections (BSIs) are frequently causes of infection, sepsis, or septic shock in hospitalized [1] and critically ill patients [2]. Whether community or hospital acquired, BSIs may complicate patients’ hospital stay and have been associated with negative outcomes [1]. The COVID-19 pandemic has caused the hospitalization of a substantial number of patients with acute respiratory failure. Previous reports have described the occurrence of BSIs in cohorts of patients with other viral pneumonia (e.g., influenza) [3,4]. The clinical features of SARS-CoV-2 infection, the use of immunomodulatory drugs, and the high rate of admission to ICU may pose patients with COVID-19 at a high risk of developing superinfections [5], such as ventilator-associated pneumonia and BSIs [6]. To date, fragmented data are available on the occurrence of BSIs in populations of hospitalized patients with COVID-19, and little is known about how BSIs may influence the outcome of these patients.

The aim of this systematic review and meta-analysis is to estimate the pooled occurrence of BSIs among hospitalized patients with COVID-19 and mortality of this patient population.

## 2. Materials and Methods

The protocol of this systematic review and meta-analysis was prospectively registered in the Open Science Framework (https://osf.io/ys8kd). For the purpose of this systematic review and meta-analysis, a systematic search was performed on PubMed, EMBASE, and Web of Science from inception to 19 April 2021. The search strategy included keywords as exact phrases and subject headings, according to database syntaxes, and is available as Supplementary Material. The reference list of relevant articles was also screened (i.e., the snowballing method). Full literature search records were then screened independently and by four authors in blinded pairs (MI, BS, CF, FRC) to identify all relevant records from titles and abstracts. Studies selected as relevant were then evaluated from the full text and included if two reviewers independently agreed on their eligibility. Nonrandomized studies, both prospective and retrospective, were considered eligible when specifically addressing the occurrence of bloodstream infections (BSIs) in adult and hospitalized patients with COVID-19, defined as the proportion of patients who developed at least one BSI during the study period, which divided the cohort of the study. The occurrence rate of BSI was the primary outcome of the study. The secondary outcome was mortality of patients with BSI and COVID-19 at the longest available follow-up. Case-control studies with a predefined or fixed proportion of enrollment between infected and non-infected were excluded, due to their design, as they were not able to provide a reliable estimate of the occurrence of BSI. Studies presenting data on only one family or species of microorganism were excluded, as they were not able to provide a reliable estimate of the overall occurrence of BSI. Studies including less than 10 patients (i.e., case series), case reports, abstracts, not peer-reviewed articles, and articles not in the English language were excluded. The Preferred Reporting Items for Systematic reviews and Meta-Analyses (PRISMA) [7] checklist is provided in Supplementary Material. Data collection was performed in duplicate and using an electronic standardized data extraction form. Discrepancies at any stage are resolved by discussion. When the disagreement was due to a difference in interpretation, arbitration was conducted by another author (GC or CM). The corresponding authors of the included studies were contacted to obtain additional information regarding eligibility or data presentation, if needed, by two authors (MI and AC).

### 2.1. Qualitative Analysis

Qualitative analysis of included studies was performed using the Methodological Index for Non-Randomized studies (MINORS) [8], due to its ability to be used for the assessment of single arm studies, independently and in duplicate by two authors (GC, MI). Disagreements over the assessment were resolved by a third author (AC). The items were scored as 0 (not reported), 1 (reported but inadequate), or 2 (reported and adequate). The global ideal score was 16 for non-comparative studies and 24 for comparative studies.

### 2.2. Statistical Analysis

Meta-analysis was performed in the case of two or more included studies reporting data on the outcomes of interest. The summary estimates were derived from logit transformation of individual study proportions of the outcomes and presented along with the corresponding 95% confidence interval (CI), calculated using random-effect meta-analysis. The I-squared (I^2^) statistical model was used to describe the percentage of variation across the included studies due to heterogeneity. Prespecified subgroup analyses were performed on the basis of the setting (e.g., intensive care unit, non-intensive care wards) and the number of centers per study (e.g., multicenter, single center). We also performed a sensitivity analysis, including studies comparing the outcomes of patients with COVID-19 to those of patients without COVID-19. A post-hoc subgroup analysis was performed on studies including only hospital-acquired BSI. All the analyses were performed by MI with inputs from AC, using Open Meta-Analyst 8 [9].

## 3. Results

### 3.1. Characteristics of Included Studies and Patients

**Figure 1:**
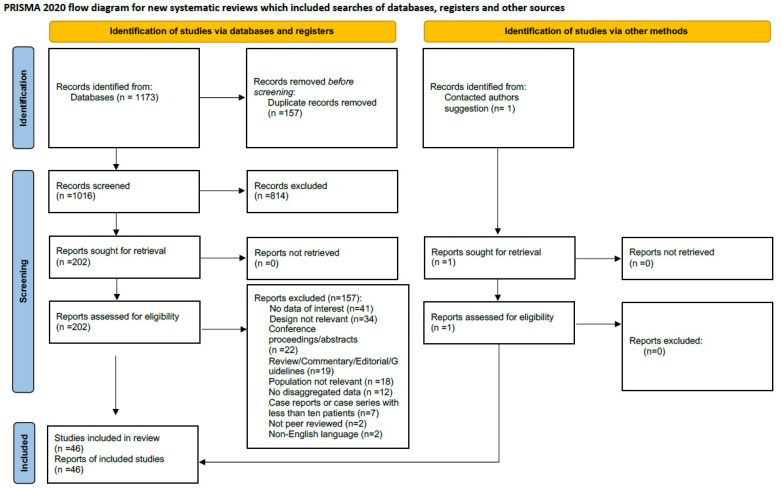
PRISMA 2020 flow diagram.

A total of 1172 records were retrieved. The full search output is available as Supplementary Material. After the screening of the records, a total of 46 studies were included in this systematic review and meta-analysis [6,10,11,12,13,14,15,16,17,18,19,20,21,22,23,24,25,26,27,28,29,30,31,32,33,34,35,36,37,38,39,40,41,42,43,44,45,46,47,48,49,50,51,52,53,54]. The included studies provided data on a total of 42,694 patients with COVID-19. The inclusion/exclusion process is presented as a PRISMA flow diagram, as shown in Figure 1.

The characteristics of the included studies are presented in Table 1. Sixteen studies were multicentric and 30 were conducted in a single center. Twenty-nine studies were conducted in EU, 10 in USA, four in China, and three in other countries. The included patients had an age ranging from 32 to 70 years, with a percentage of male gender in the cohorts ranging from 48% to 94%. All the studies were single-arm studies, except 11 also presenting data on patients without COVID-19 as a comparator. Details on isolates and source of the infection are provided in Table S2, Supplementary Material. Data on causative microorganisms showed that the infections were mainly bacterial and rarely fungal BSIs, with Gram positive species prevalent in many studies. The qualitative assessment per study, according to MINORS, is provided in Tables S3 and S4, Supplementary Material. Only two [52,55] of the studies reported protocol registration and none reported information on sample calculation. Among the 11 studies with a comparison group, all except two had historical comparison groups [27,35]. Furthermore, 8 out of 11 studies reported only unadjusted statistical analysis [10,12,18,20,22,27,28,44]. These were the most frequently downgraded domains.

### 3.2. Outcomes

**Figure 2:**
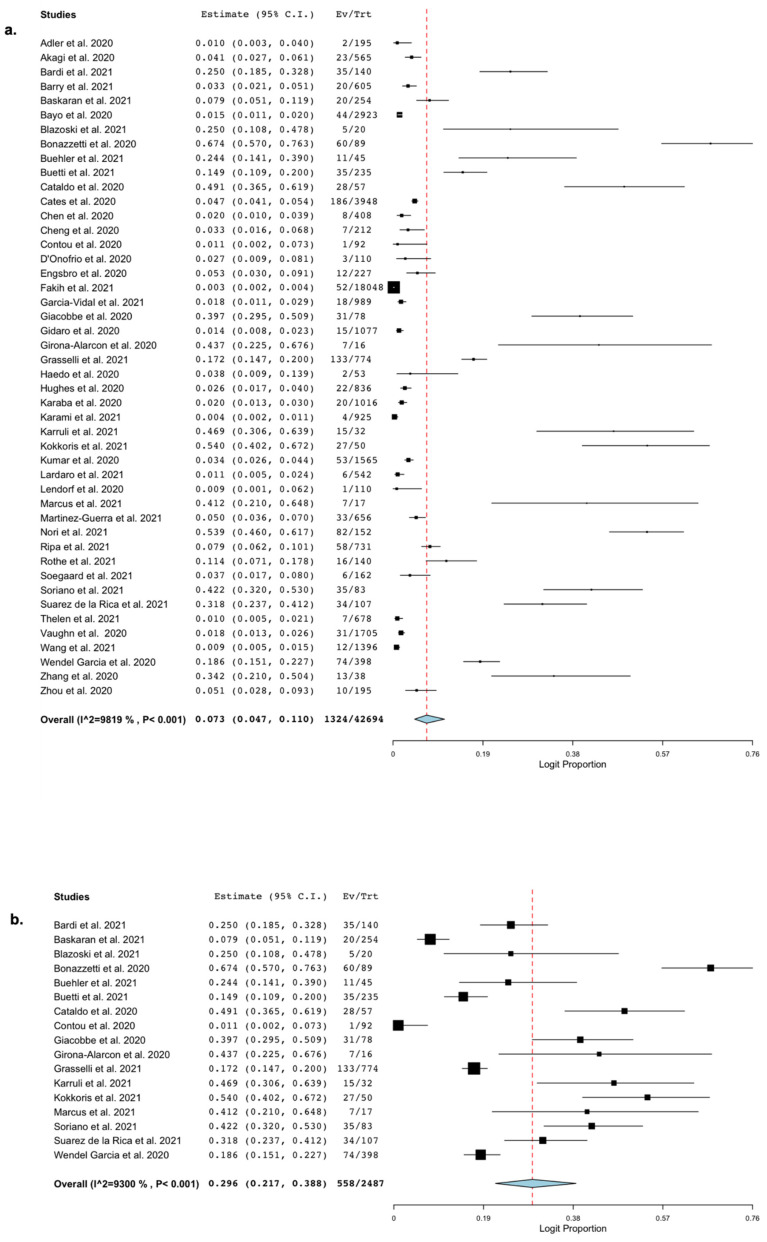
Occurrence of bloodstream infection. Forest plot with the result of single-arm meta-analysis for the occurrence of BSI in patients with COVID-19. BSI, bloodstream infection; CI, confidence interval; Ev, events; Trt, total. (**a**) Pooled estimated occurrence of bloodstream infection among hospitalized patients with COVID-19. (**b**) Pooled estimated occurrence of bloodstream infection among patients with COVID-19 admitted to ICU.

All the included studies reported the occurrence of BSI in cohorts of hospitalized patients with COVID-19. The pooled estimated occurrence of BSI was 7.3% (95% CI 4.7–1.1%; 1324/42,694 patients; I^2^ = 98%; Figure 2a).

**Figure 3:**
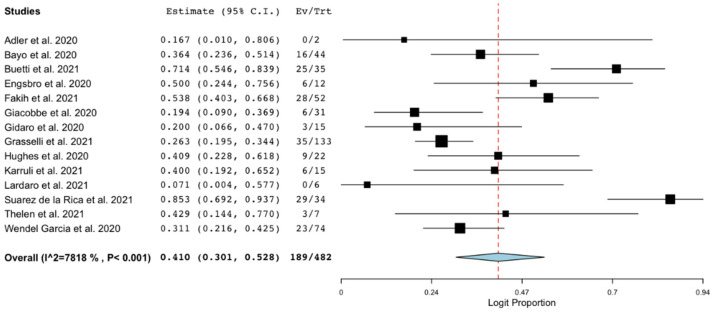
Mortality of patients with COVID-19 and bloodstream infection. Forest plot with the result of single-arm meta-analysis for mortality of patients with COVID-19 and BSI. BSI, bloodstream infection; CI, confidence interval; Ev, events; Trt, total.

Fourteen studies [6,10,18,20,27,28,32,34,35,38,42,46,50,55] reported data on mortality in patients with COVID-19 and BSI. The pooled estimated occurrence of mortality (at the longest available follow-up) was 41% (95% CI 30%–52.8%; 189/482 patients; I^2^ = 78%; Figure 3).

### 3.3. Sensitivity and Subgroup Analyses

The pooled estimate of the occurrence of BSI in patients admitted to ICUs was 29.6% (95% CI 21.7%–38.8%; 558/2487 patients; I^2^ = 93%; Figure 2b).

Concerning the occurrence of BSI based on the number of centers, the pooled estimate was 4.7% (95% CI 2.5–8.7%; 670/14,169 patients; I^2^ = 98%; Figure S1, Supplementary Material) when considering only multicenter studies and 9.1% (95% CI 4.9–16.5%; 654/28,525 patients; I^2^ = 98%; Figure S2, Supplementary Material) when considering only single center studies.

In the post-hoc subgroup analysis, considering only data from studies specifically addressing hospital-acquired BSI, the pooled estimate of the occurrence of BSI was 12.2% (95% CI 6.6%–21.3%; 606/9839 patients; I^2^ = 98%; Figure S3, Supplementary Material).

**Figure 4:**
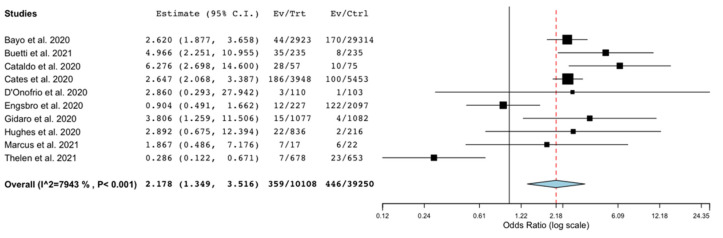
Occurrence of bloodstream infection in patients with COVID-19 compared with patients without COVID-19. Forest plot with the results of the sensitivity analysis on the occurrence of BSI in patients with COVID-19 compared to patients without COVID-19. BSI, bloodstream infection; CI, confidence interval; Ctrl, controls; Ev, events; Trt, total.

We also conducted a sensitivity analysis considering the occurrence of BSI patients without COVID-19, in comparison with those with COVID-19. Eleven studies provided data on a comparison group, but one study [18] did not provide the denominator to calculate the occurrence. Thus, in this analysis, 10 studies [6,10,12,16,20,22,27,28,35,44] providing unadjusted data on occurrence of BSI in the comparison group were included. There was found a significantly higher risk of BSI in patients with COVID-19 compared to patients without COVID-19 (OR 2.18; 95% CI 1.35–3.51; *p* < 0.001; I^2^ = 79%; Figure 4).

## 4. Discussion

To the best of our knowledge, this is the most updated systematic review and meta-analysis estimating the occurrence of BSIs among hospitalized patients with COVID-19. Our main finding was that around 7% of hospitalized patients with COVID-19 may experience a BSI. This data was comparable with that measured among hospitalized patients prior to the pandemic [56]. Interestingly, around one out of three patients with COVID-19, once admitted to ICU, may have a clinical course complicated by a BSI. This finding is also in line with the previously estimated prevalence of BSIs in adult patients with sepsis or septic shock [1,57] and almost double than the prevalence of BSIs in cohorts of adult patients admitted to ICU [58].

A higher occurrence of BSI in patients with COVID-19 was also observed, in comparison with patients without COVID-19 (OR 2.77; 95% CI 1.53–5.02; *p* < 0.001). Although caution is needed considering this result, coming from an unadjusted analysis, the development of a BSI in patients with COVID-19 may share some risk factors with other superinfections, frequently observed in patients with COVID-19 [5,59]. Hospital and ICU overcrowding, a difficult application of infection prevention strategies while using PPE in such settings, patients’ immunological impairment, and the frequent need for central lines can be counted as risk factors specifically belonging to patients with COVID-19.

During the early phases of the pandemic, many centers supported the protocolized administration of antibiotic therapy at the time of diagnosis or hospital admission due to COVID-19. Considering our data, in addition to what was already issued on the topic [60], that approach may not be probably justified by the real occurrence of superinfections, and a tailored approach is needed. A clinical suspect should still probably remain the condictio sine qua non to consider the beginning of an antibiotic therapy in patients with COVID-19, taking into account that those admitted to ICU may deserve specific attention.

Our study has strengths, such as the comprehensive search, the methodology, and the reporting according to PRISMA 2020 [7], the large number of studies and patients evaluated, and the geographical variety of the settings. Moreover, incompletely reported data were retrieved from the corresponding authors when feasible. Our analysis also has limitations. The nature of this study was descriptive, and no analysis was performed to look for associations with potential risk factors. Our analysis also had a high statistical heterogeneity. The high statistical heterogeneity across the studies may be attributed to the different criteria used to include patients, based on the origin of the infection (i.e., community-acquired, hospital-acquired, ICU-acquired), the variable sampling rate, or the different settings (i.e., ICU or non-ICU wards). Other factors, such as patients’ characteristics or clinical severity may also explain heterogeneity. However, we tried to reduce the impact of heterogeneity with several pre-planned sensitivity and subgroup analyses. Data on causative microorganisms were also heterogeneously reported across the studies; thus, we decided to report them as per the authors’ descriptions, and we did not perform any quantitative analysis on causative microorganisms.

## 5. Conclusions

A total of 7% of hospitalized patients with COVID-19 may experience BSI with a mortality rate of around 40%. Almost 30% of patients admitted to the ICU may develop BSI during their ICU stay. Hospitalized patients with COVID-19 may have three-times higher odds of developing BSI than patients without COVID-19.

## Figures and Tables

**Table 1:** Characteristics of the included studies. The table shows the main characteristics of the included studies, as reported by the authors. BSI, bloodstream infection; CRBI, catheter-related bloodstream infection; ECMO, extracorporeal membrane oxygenation; ICU, intensive care unit; NA, not available; RT-PCR, real-time polymerase chain reaction.

Authors (Year)	Design of the Study (Country)	Setting	Population	Comparison Group (non COVID-19):	Definitions
Adler et al. (2020) [38]	Single center retrospective observational study (UK)	Both ICU and non-ICU	195 hospitalized patients with COVID-19 (RT-PCR); Age 69 y. [59–81]; Male 60.5%	NA	Samples unequivocally consistent with contamination were considered negative. Results recorded until 7 days from the positive COVID-19 to exclude hospital-acquired infections
Akagi et al. (2021) [45]	Single center retrospective, case–control study (USA)	Non-ICU	565 hospitalized patients with COVID-19; Age 64.5 y. ±16.4; Male 57.1%	NA	Community onset bloodstream infection Positive blood culture with a known pathogen in one or more blood cultures or the same commensal organism in two or more blood cultures drawn within 48 h of hospitalization
Bardi et al. (2021) [14]	Single center retrospective observational study (Spain)	ICU	140 patients with severe COVID-19 (RT-PCR), admitted to ICU Age 61 y. [57–67], Male 77%	NA	All infections were defined according to the Centers for Disease Control and Prevention criteria and the Spanish Society of Infectious Diseases and Clinical Microbiology
Barry et al. (2021) [49]	Single center case series (Saudi Arabia)	Both ICU and non-ICU	605 hospitalized patients with COVID-19 Age 75% < 65 y. Male 61%	NA	NA
Baskaran et al. (2021) [33]	Multicenter retrospective observational study (UK)	ICU	254 patients with COVID-19 (RT-PCR) admitted to ICU, Age 59 y. [49–69], Male 64.6%	NA	Culture results were excluded if they were considered to represent contamination or colonisation
Bayo et al. (2020) [28]	Single center retrospective cohort study (Spain)	Both ICU and non-ICU	2923 hospitalized patients with COVID-19 (RT-PCR), Age 64.5 [NA] Male 86%	29,314 hospitalized patients, Age 65.9 [NA] Male 67%	Blood culture contamination was defined as the presence of one or more of the following organisms found in only one blood culture set and only one of a series of two or three blood culture sets: coagulase-negative staphylococci, Micrococcus spp., viridans group streptococci, Propionibacterium acnes, Corynebacterium spp., and Bacillus spp.
Blazoski et al. (2021) [39]	Single center retrospective observational study (USA)	ICU	20 patients with COVID-19 admitted to ICU and treated with ECMO; Age 54 ± 8.7 Male 60%	NA	NA
Bonazzetti et al. (2020) [36]	Single center retrospective observational study (Italy)	ICU	89 patients with COVID-19 admitted to ICU, Age 61.5 y. [53.1–68.7]; Male 77.5%	NA	BSIs were defined using the Center for Disease and Control criteria. BSI due to organisms usually associated with contamination had to be confirmed in two sets of blood cultures. ICU-acquired BSI if diagnosed greater than or equal to 48 h after ICU admission
Buehler et al. (2021) [37]	Single center prospective observational study (Switzerland)	ICU	45 patients with COVID-19 (RT-PCR) admitted to ICU, Age 60 y. [54–69], Male 77.8%	NA	NA
Buetti et al. (2021) [6]	Multicenter prospective observational study (France)	ICU	235 patients with COVID-19, admitted to ICU, Age 59.8 y. ± 12.7 Male 80%	Historical cohort of 235 patients with ARDS, admitted to ICU Age 59.8 y. ± 13.8 Male 80%	ICU-BSI infection onset occurring >48 h after ICU admission. Typical skin contaminants were included if ≥2 blood cultures showed the same phenotype within a 48-h period or ≥1 blood culture positive for clinical sepsis, no other infectious process, and antibacterial agent treatment initiated by the attending physician Secondary BSI same microorganism in one blood culture and in the suspected source of infection. All catheter-related BSI (CRBSI) were documented by quantitative tip culture
Cataldo et al. (2020) [12]	Single center retrospective cohort study (Italy)	ICU	57 patients with COVID-19 admitted to ICU, Age 62 y. ± 13, Male 72%	Historical cohort of 75 patients admitted to ICU	NA
Cates et al. (2020) [16]	Multicenter retrospective observational study (USA)	Both ICU and non-ICU	3948 hospitalized patients with COVID-19 (RT-PCR), Age 70 y. [61–77], Male 94%	5453 hospitalized patients with influenza A or B (RT-PCR or other), Age 69 [61–75], Male 93.8%	ICD-10-CM codes
Chen et al. (2020) [30]	Single center retrospective observational study (China)	Both ICU and non-ICU	408 hospitalized patients with COVID-19 (PCR), Age 48 y. [34–60], Male 48%	NA	BSI non-skin flora commensal on one or more blood culture to define a bloodstream infection as that caused by a common skin colonizer. Two or more blood cultures drawn from different sites were required plus a clinical evaluation. Co-infections if present at the time of admission (initial 48 h), secondary infections if emerged during the course of hospitalization
Cheng et al. (2020) [47]	Single center retrospective observational study (China)	NA	212 hospitalized patients with COVID-19; Age 53.1 y. ± 16.6; Male 60%	NA	According to the diagnostic standard of nosocomial infection formulated by the China’s Ministry of Health in 2001
Contou et al. (2020) [53]	Single center retrospective observational study (France)	ICU	92 patients with COVID-19 (RT-PCR) admitted to ICU, Age 61 y. [55–70], Male 79%	NA	A patient was considered as co-infected when at least one of the performed microbiological investigations isolated a pathogenic bacterium
D’Onofrio et al. (2020) [22]	Single center prospective and retrospective observational study (Belgium)	Both ICU and non-ICU	110 patients hospitalized with COVID-19 (RT-PCR), Age 73 y. [60–82], Male 63%	103 hospitalized patients with influenza, Age 76 y. [57–84], Male 51%	Blood cultures were drawn at admission (<24 h)
Engsbro et al. (2020) [27]	Single center prospective observational cohort study (Denmark)	Both ICU and non-ICU	227 hospitalized patients with COVID-19 (RT-PCR); Age 66.3 y. ± 17, Male 51%	2097 hospitalized patients	Bloodstream infections were categorized as community-acquired or hospital-acquired if cultures were drawn within or after 48 h of admission. Clinical significance assessed by microbiologist
Fakih et al. (2021) [18]	Retrospective study (USA)	Both ICU and non-ICU	18,048 hospitalized patients with COVID-19	Hospitalized patients	NA
Garcia-Vidal et al. (2021) [43]	Single center observational cohort study (Spain)	Both ICU and non-ICU	989 hospitalized adults with COVID-19 (RT- PCR), tested for BSI Age 62 y. [48–74], Male 56%	NA	BSI Non-skin flora commensal on one or more blood culture or common skin colonizer in two or more blood cultures from different sites, plus clinical suspect. Community-acquired diagnosis within the first 24 h of admission Hospital-acquired >48 h after admission
Giacobbe et al. (2020) [50]	Single center retrospective observational study (Italy)	ICU	78 patients with COVID-19 (RT-PCR) admitted to ICU, Age 66 y. [57–70], Male 77%	NA	ICU-acquired BSI At least one positive blood culture for bacteria or fungi, drawn at >48 h after ICU admission. For other common skin contaminants, at least two consecutive blood cultures were positive for the same pathogen. In patients with multiple blood cultures that were positive for the same organism, novel BSI events were considered as independent if occurring at least 30 days after the previous event. Polymicrobial infections were considered as separate BSI events, one for each causative organism isolated from the blood culture
Gidaro et al. (2020) [35]	Multicenter retrospective cohort study (Italy)	Both ICU and non-ICU	1077 hospitalized patients with COVID-19	1082 hospitalized patients	CRBSI diagnosed with blood culture performed by the catheter that showed microbial growth at least 2 h earlier than growth detected in blood collected simultaneously from a peripheral vein
Girona-Alarcon et al. (2020) [26]	Single center prospective observational study (Spain)	ICU	16 patients with COVID-19 (PCR) admitted to ICU Age 32 y. [23.3–41.5] Male 56.3%	NA	
Grasselli et al. (2021) [55]	Multicenter retrospective analysis of prospectively collected data (Italy)	ICU	774 patients with COVID-19 admitted to ICU; Age 62 y. [54–68] Male 77%	NA	Infections were considered as ICU acquired infections whether they occurred ≥48 h from ICU admission
Haedo et al. (2020) [24]	Single center secondary analysis of a prospective observational study (Argentina)	Both ICU and non-ICU	53 hospitalized patients with COVID-19 (RT-PCR)	NA	NA
Hughes et al. (2020) [10]	Multicenter retrospective observational study (UK)	Both ICU and non-ICU	836 hospitalized patients with confirmed SARS-CoV-2 (RT- PCR), Age 69.5 y. [55–81], Male 62%	Historical cohort of 216 hospitalized patients positive to influenza A or B Age 36 y. [22–65], Male 42%	Commensal Pathogens not warranting targeted therapy; Community acquired infection Less than 120 h from admission; Hospital acquired infection More than 120 h from admission
Karaba et al. (2020) [13]	Multicenter retrospective observational study (USA)	Both ICU and non-ICU	1016 hospitalized patients with COVID-19 (NAAT), Age 61 [48–74], Male 54%	NA	BSI Organisms recovered from blood culture and deemed not to be a contaminant. Co-infections were considered present at the time of admission (initial 48 h)
Karami et al. (2021) [17]	Multicenter retrospective observational study (The Netherlands)	Both ICU and non-ICU	925 hospitalized patients with COVID-19 (PCR), Age 70 y. [59–77], Male 64%	NA	Bacterial co-infection was defined as the isolation of a bacterium from a blood culture. Typical contaminants were excluded. The early phase was defined as the first 7 calendar days of admission.
Karruli et al. (2021) [42]	Single center retrospective observational study (Italy)	ICU	32 patients with COVID-19 admitted to ICU; Age 68 y. [55.2–75]; Male 71.9%	NA	Infections were diagnosed based on the current US Centers for Disease Control and Prevention National Health care Safety Network criteria. Multidrug resistance was defined according to the Magiorakos et al. criteria.
Kokkoris et al. (2021) [15]	Single center retrospective observational study (Greece)	ICU	50 patients with COVID-19, admitted to ICU Age 64 y. Male 76%	NA	ICU-acquired BSI pathogen isolation from ≥1 blood specimen obtained at more than 48 h after ICU admission. In patients with ≥2 BSIs, only the first BSI was included, unless the subsequent episode was fungal
Kumar et al. (2020) [41]	Single center retrospective observational study (USA)	Both ICU and non-ICU	1565 hospitalized patients with COVID-19	NA	Hospital acquired BSI if cultures were positive and obtained after 3 days of hospital admission
Lardaro et al. (2021) [34]	Multicenter retrospective observational study (USA)	Both ICU and non-ICU	542 hospitalized patients with COVID-19 (RT-PCR), Age 62.8 y. ± 16.5, Male 49.6%	NA	True positive bacteremia At least two of four bottles grew bacteria. The growth of bacteria outside of typical skin flora was generally considered true positive
Lendorf et al. (2020) [25]	Single center retrospective observational study (Denmark)	Both ICU and non-ICU	110 hospitalized patients with COVID-19 (RT-PCR) Age 68 y. (56–78) Male 60%	NA	Samples were acquired within 3 days of hospitalization. Clinically relevant if likely to contribute to symptomatology and guided treatment
Marcus et al. (2021) [44]	Single center retrospective observational study (USA)	ICU	17 patients with COVID-19 admitted to ICU and treated with ECMO; Age 42 y. [35–49], Male 76%	22 patients with influenza admitted to ICU and treated with ECMO; Age 45 y. [35–55], Male 68%	BSI Positive cultures during ECMO course or within 48 h of decannulation that were determined to be pathogenic by the patient’s treatment team
Martinez-Guerra et al. (2021) [48]	Single center prospective cohort study (Mexico)	Both ICU and non-ICU	794 hospitalized patients with COVID-19 (RT-PCR) (data presented on 656 patients with complete follow-up) Age 52 y. [43–62] Male 61.6%	NA	NA
Nori et al. (2021) [54]	Multicenter retrospective observational study (USA)	Both ICU and non-ICU	152 hospitalized patients with COVID-19 (PCR) Age 62 y. [52.5–72] Male 59%	NA	All cases were reviewed by an infectious diseases specialist to determine the presence of true clinical coinfection and the source. National Healthcare Safety Network criteria were used for central-line–associated bloodstream infections
Ripa et al. (2021) [52]	Single center prospective observational study (Italy)	Both ICU and non-ICU	731 hospitalized patients with COVID-19 (RT-PCR), Age 64 [55–76], Male 67.9%	NA	BSI Single positive blood culture for a likely pathogen or two or more positive blood cultures for common skin colonizers without a concomitant microbiologically documented lower respiratory tract infection due to the same pathogen. Patients with more than one positive blood culture within 7 days from the first positive blood culture were considered to have a single episode of BSI with multiple isolates
Rothe et al. (2021) [40]	Single center retrospective observational study (Germany)	Both ICU and non-ICU	140 hospitalized patients with COVID-19 (RT-PCR or serological); Age 63.5 y. (range 17–99); Male 64%	NA	In case of coagulase-negative staphylococci, the isolates were considered clinically significant (true bacteraemia) if two or more bottles yielded the same microorganism.
Søgaard et al. (2021) [29]	Single center retrospective observational study (Switzerland)	Both ICU and non-ICU	162 hospitalized patients with COVID-19 (RT-PCR), Age 64.4 y. [50.4–74.2] Male 61.1%	NA	Community-acquired bloodstream infection pathogen from a blood culture taken within 48 h of hospitalization. Hospital-acquired bloodstream infection pathogen from a blood culture taken 48 h or more after hospitalization.
Soriano et al. (2021) [31]	Single center restrospective observational study (Spain)	ICU	83 patients with COVID-19 (RT-PCR) admitted to ICU, Age 61.2 y. ± 10.4, Male 79.5%	NA	NA
Suarez de la Rica et al.(2021) [32]	Single center retrospective study (Spain)	ICU	107 patients with COVID-19 (RT-PCR), admitted to ICU, Age 62.2 y. ± 10.6, Male 71%	NA	Nosocomial bacteremia positive blood cultures recovered at least 48 h after the hospital admission. Coagulase-negative staphylococci considered as contaminants (only one positive blood culture) were excluded
Thelen et al. (2021) [20]	Multicenter retrospective cohort study (The Netherlands)	Both ICU and non-ICU	678 hospitalized patients with COVID-19 (RT-PCR), Age 70 y. [58–78], Male 65%	653 patients with influenza A or B (RT-PCR)	Bacteria were categorized as likely contaminants if they were affiliated to groups that represent commensal skin microbiota and were defined in the patient’s medical record as a contaminant by the Department of Medical Microbiology. Blood cultures were collected within a time interval of 48 h before and after the RT-PCR test
Vaughn et al. (2020) [11]	Multicenter retrospective observational study (USA)	Both ICU and non-ICU	1705 hospitalized patients with COVID-19, Age y. 64.7 [53–76.7], Male 52%	NA	Community onset bacterial coinfections were identified by blood culture positive for a typically pathogenic bacterium
Wang et al. (2021) [19]	Multicenter retrospective observational cohort study (UK)	Both ICU and non-ICU	1396 hospitalized patients with COVID-19 (RT-PCR), Age 67.4 y. ± 16.2, Male 64.7%	NA	Microbiological specimens taken within 48 h of admission. Two senior consultant microbiologists reviewed the clinical significance of the test results and the likelihood of contamination or colonization based on the nature of the isolated organisms
Wendel Garcia et al. (2020) [46]	Multicenter prospective observational cohort study (EU)	ICU	639 patients with COVID-19 admitted to ICU (data presented on 398 patients who reached the outcome of discharged/dead) Age 63 [53–71]; Male 75%	NA	Bacteraemia and fungaemia were defined as positive blood cultures for a bacterial or fungal pathogen
Zhang et al. (2020) [51]	Multicenter retrospective cohort study (China)	Both ICU and non-ICU	38 patients with severe or critical COVID-19, Age 64.76 y. ± 13.76, Male 84%	NA	Secondary infection clinical symptoms or positive radiologic evidence and a positive laboratory-confirmed aetiologic result (culture positive or mNGS positive confirming by RT–PCR) after 48 h of admission. The final diagnosis of causative agents was made according to the clinical physician expert groups’ discussion results
Zhou et al. (2020) [23]	Multicenter retrospective observational study (China)	Both ICU and non-ICU	195 patients with COVID-19 (RT-PCR), admitted to ICU, Age 66 y. [56–76], Male 66.7%	NA	NA

## Data Availability

The data presented in this study are available in Supplementary Materials. The unpublished data retrieved by private correspondence with the authors and used in this study analysis are available on request from the corresponding author.

## Supplementary Materials

The following are available online at https://www.mdpi.com/article/10.3390/microorganisms9102016/s1, PDF S1: Search strategy; PDF S2: Figure S1: Forest plot with the results of the subgroup analysis conducted on the occurrence of bloodstream infections in patients with COVID-19 in multicenter studies; Figure S2: Forest plot with the results of the subgroup analysis conducted on the occurrence of bloodstream infections in patients with COVID-19 in single center studies; Figure S3: Forest plot with the result of the post-hoc subgroup analysis, considering only data from studies specifically addressing hospital-acquired bloodstream infections; Table S1: PRISMA Main Checklist and PRISMA Abstract Checklist; Table S2: Characteristics of bloodstream infections and isolates, Table S3: Quality assessment of studies according to MINORS score for non-comparative studies; Table S4: Quality assessment of studies according to MINORS additional score for comparative studies; Excel S1: Full search output.

## References

[1] Timsit J.F., Ruppé E., Barbier F., Tabah A., Bassetti M. (2020). Bloodstream infections in critically ill patients: An expert statement. Intensive Care Med..

[2] Russotto V., Cortegiani A., Graziano G., Saporito L., Raineri S.M., Mammina C., Giarratano A. (2015). Bloodstream infections in intensive care unit patients: Distribution and antibiotic resistance of bacteria. Infect. Drug Resist..

[3] Blyth C.C., Webb S.A.R., Kok J., Dwyer D.E., van Hal S.J., Foo H., Ginn A.N., Kesson A.M., Seppelt I., Iredell J.R. (2013). The impact of bacterial and viral co-infection in severe influenza. Influenza Other Respir. Viruses.

[4] Martín-Loeches I., Sanchez-Corral A., Diaz E., Granada R.M., Zaragoza R., Villavicencio C., Albaya A., Cerdá E., Catalán R.M., Luque P. (2011). Community-acquired respiratory coinfection in critically III patients with pandemic 2009 influenza A(H1N1) virus. Chest.

[5] Ippolito M., Misseri G., Catalisano G., Marino C., Ingoglia G., Alessi M., Consiglio E., Gregoretti C., Giarratano A., Cortegiani A. (2021). Ventilator-associated pneumonia in patients with covid-19: A systematic review and meta-analysis. Antibiotics.

[6] Buetti N., Ruckly S., de Montmollin E., Reignier J., Terzi N., Cohen Y., Shiami S., Dupuis C., Timsit J.F. (2021). COVID-19 increased the risk of ICU-acquired bloodstream infections: A case–cohort study from the multicentric OUTCOMEREA network. Intensive Care Med..

[7] Page M., McKenzie J., Bossuyt P., Boutron I., Hoffmann T., Mulrow C., Shamseer L., Tetzlaff J.M., Akl E.A., Brennan S.E. (2021). The PRISMA 2020 statement: An updated guideline for reporting systematic reviews. BMJ.

[8] Slim K., Nini E., Forestier D., Kwiatkowski F., Panis Y., Chipponi J. (2003). Methodological index for non-randomized studies (Minors): Development and validation of a new instrument. ANZ J. Surg..

[9] Wallace B.C., Dahabreh I.J., Trikalinos T.A., Lau J., Trow P., Schmid C.H. (2012). Open Meta Analyst; Closing the gap between methodologists and end-users: R as a computational back-end. Stat. Softw..

[10] Hughes S., Troise O., Donaldson H., Mughal N., Moore L.S.P. (2020). Bacterial and fungal coinfection among hospitalized patients with COVID-19: A retrospective cohort study in a UK secondary-care setting. Clin. Microbiol. Infect..

[11] Vaughn V.M., Gandhi T.N., Petty L.A., Patel P.K., Prescott H.C., Malani A.N., Ratz D., McLaughlin E., Chopra V., Flanders S.A. (2021). Empiric Antibacterial Therapy and Community-onset Bacterial Coinfection in Patients Hospitalized With Coronavirus Disease 2019 (COVID-19): A Multi-hospital Cohort Study. Clin. Infect. Dis..

[12] Cataldo M.A., Tetaj N., Selleri M., Marchioni L., Capone A., Caraffa E., Di Caro A., Petrosillo N. (2020). Incidence of bacterial and fungal bloodstream infections in COVID-19 patients in intensive care: An alarming “collateral effect”. J. Glob. Antimicrob. Resist..

[13] Karaba S.M., Jones G., Helsel T., Smith L.L., Avery R., Dzintars K., Salinas A.B., Keller S.C., Townsend J.L., Klein E. (2021). Prevalence of co-infection at the time of hospital admission in COVID-19 Patients, A multicenter study. Open Forum Infect. Dis..

[14] Bardi T., Pintado V., Gomez-Rojo M., Escudero-Sanchez R., Azzam Lopez A., Diez-Remesal Y., Martinez Castro N., Ruiz-Garbajosa P., Pestaña D. (2021). Nosocomial infections associated to COVID-19 in the intensive care unit: Clinical characteristics and outcome. Eur. J. Clin. Microbiol. Infect. Dis..

[15] Kokkoris S., Papachatzakis I., Gavrielatou E., Ntaidou T., Ischaki E., Malachias S., Vrettou C., Nichlos C., Kanavou A., Zervakis D. (2021). ICU-acquired bloodstream infections in critically ill patients with COVID-19. J. Hosp. Infect..

[16] Cates J., Lucero-Obusan C., Dahl R.M., Schirmer P., Garg S., Oda G., Hall A.J., Langley G., Havers F.P., Holodniy M. (2020). Risk for In-Hospital Complications Associated with COVID-19 and Influenza—Veterans Health Administration, United States, October 1, 2018–May 31, 2020. MMWR Morb. Mortal. Wkly. Rep..

[17] Karami Z., Knoop B.T., Dofferhoff A.S.M., Blaauw M.J.T., Janssen N.A., van Apeldoorn M., Kerckhoffs A.P.M., van de Maat J.S., Hoogerwerf J.J., ten Oever J. (2021). Few bacterial co-infections but frequent empiric antibiotic use in the early phase of hospitalized patients with COVID-19: Results from a multicentre retrospective cohort study in The Netherlands. Infect. Dis. (Auckl.).

[18] Fakih M.G., Bufalino A., Sturm L., Huang R.H., Ottenbacher A., Saake K., Winegar A., Fogel R., Cacchione J. (2021). COVID-19 Pandemic, CLABSI, and CAUTI: The Urgent Need to Refocus on Hardwiring Prevention Efforts. Infect. Control Hosp. Epidemiol..

[19] Wang L., Amin A.K., Khanna P., Aali A., Mcgregor A., Bassett P., Gopal Rao G. (2021). An observational cohort study of bacterial co-infection and implications for empirical antibiotic therapy in patients presenting with COVID-19 to hospitals in North West London. J. Antimicrob. Chemother..

[20] Thelen J.M., (Noud) Buenen A.G., van Apeldoorn M., Wertheim H.F., Hermans M.H.A., Wever P.C. (2021). Community-acquired bacteraemia in COVID-19 in comparison to influenza A and influenza B: A retrospective cohort study. BMC Infect. Dis..

[21] Grasselli G., Zangrillo A., Zanella A., Antonelli M., Cabrini L., Castelli A., Cereda D., Coluccello A., Foti G., Fumagalli R. (2020). Baseline Characteristics and Outcomes of 1591 Patients Infected with SARS-CoV-2 Admitted to ICUs of the Lombardy Region, Italy. JAMA.

[22] D’Onofrio V., Van Steenkiste E., Meersman A., Waumans L., Cartuyvels R., Van Halem K., Messiaen P., Gyssens I.C. (2021). Differentiating influenza from COVID-19 in patients presenting with suspected sepsis. Eur. J. Clin. Microbiol. Infect. Dis..

[23] Zhou S., Yang Y., Zhang X., Li Z., Liu X., Hu C., Chen C., Wang D., Peng Z. (2020). Clinical Course of 195 Critically Ill COVID-19 Patients: A Retrospective Multicenter Study. Shock.

[24] Haedo M., Melendi S., Mauri M., Ujeda C., Leis R. (2020). Usefulness of blood coltures in COVID-19 pneumonia. Medicina.

[25] Lendorf M., Boisen M., PL K., Lokkegard E., Krog S., Brandi L., Fischer T. (2020). Characteristics and early outcomes of patients hospitalised for COVID-19 in North Zealand, Denmark. Dan. Med. J..

[26] Girona-Alarcon M., Bobillo-Perez S., Sole-Ribalta A., Hernandez L., Guitart C., Suarez R., Balaguer M., Cambra F.J., Jordan I. (2021). The different manifestations of COVID-19 in adults and children: A cohort study in an intensive care unit. BMC Infect. Dis..

[27] Engsbro A.L., Israelsen S.B., Pedersen M., Tingsgaard S., Lisby G., Andersen C., Benfield T. (2020). Predominance of hospital-acquired bloodstream infection in patients with Covid-19 pneumonia. Infect. Dis. (Auckl.).

[28] Mormeneo Bayo S., Palacián Ruíz M.P., Moreno Hijazo M., Villuendas Usón M.C. (2021). Bacteremia during COVID-19 pandemic in a tertiary hospital in Spain. Enferm. Infecc. Microbiol. Clin..

[29] Søgaard K.K., Baettig V., Osthoff M., Marsch S., Leuzinger K., Schweitzer M., Meier J., Bassetti S., Bingisser R., Nickel C.H. (2021). Community-acquired and hospital-acquired respiratory tract infection and bloodstream infection in patients hospitalized with COVID-19 pneumonia. J. Intensive Care.

[30] Chen S., Zhu Q., Xiao Y., Wu C., Jiang Z., Liu L., Qu J. (2021). Clinical and etiological analysis of co-infections and secondary infections in COVID-19 patients: An observational study. Clin. Respir. J..

[31] Soriano M.C., Vaquero C., Ortiz-Fernández A., Caballero A., Blandino-Ortiz A., de Pablo R. (2021). Low incidence of co-infection, but high incidence of ICU-acquired infections in critically ill patients with COVID-19. J. Infect..

[32] Suarez-de-la- A., Falces-romero I. (2021). Original Secondary infections in mechanically ventilated patients with COVID-19: An overlooked matter ?. Rev. Española Quimioter..

[33] Baskaran V., Lawrence H., Lansbury L.E., Webb K., Safavi S., Zainuddin N.I., Huq T., Eggleston C., Ellis J., Thakker C. (2021). Co-infection in critically ill patients with COVID-19: An observational cohort study from England. J. Med. Microbiol..

[34] Lardaro T., Wang A.Z., Bucca A., Croft A., Glober N., Holt D.B., Musey P.I., Peterson K.D., Trigonis R.A., Schaffer J.T. (2021). Characteristics of COVID-19 patients with bacterial coinfection admitted to the hospital from the emergency department in a large regional healthcare system. J. Med. Virol..

[35] Gidaro A., Vailati D., Gemma M., Lugli F., Casella F., Cogliati C., Canelli A., Cremonesi N., Monolo D., Cordio G. (2021). Retrospective survey from vascular access team Lombardy net in COVID-19 era. J. Vasc. Access.

[36] Bonazzetti C., Morena V., Giacomelli A., Oreni L., Casalini G., Galimberti L.R., Bolis M., Rimoldi M., Ballone E., Colombo R. (2020). Unexpectedly High Frequency of Enterococcal Bloodstream Infections in Coronavirus Disease 2019 Patients Admitted to an Italian ICU: An Observational Study. Crit. Care Med..

[37] Buehler P.K., Zinkernagel A.S., Hofmaenner D.A., Wendel Garcia P.D., Acevedo C.T., Gómez-Mejia A., Mairpady Shambat S., Andreoni F., Maibach M.A., Bartussek J. (2021). Bacterial pulmonary superinfections are associated with longer duration of ventilation in critically ill COVID-19 patients. Cell Rep. Med..

[38] Adler H., Ball R., Fisher M., Mortimer K., Vardhan M.S. (2020). Low rate of bacterial co-infection in patients with COVID-19. Lancet Microbe.

[39] Blazoski C., Baram M., Hirose H. (2021). Outcomes of extracorporeal membrane oxygenation in acute respiratory distress syndrome due to COVID-19: The lessons learned from the first wave of COVID-19. J. Card. Surg..

[40] Rothe K., Feihl S., Schneider J., Wallnöfer F., Wurst M., Lukas M., Treiber M., Lahmer T., Heim M., Dommasch M. (2021). Rates of bacterial co-infections and antimicrobial use in COVID-19 patients: A retrospective cohort study in light of antibiotic stewardship. Eur. J. Clin. Microbiol. Infect. Dis..

[41] Kumar G., Adams A., Hererra M., Rojas E.R., Singh V., Sakhuja A., Meersman M., Dalton D., Kethireddy S., Nanchal R. (2021). Predictors and outcomes of healthcare-associated infections in COVID-19 patients. Int. J. Infect. Dis..

[42] Karruli A., Boccia F., Gagliardi M., Patauner F., Ursi M.P., Sommese P., De Rosa R., Murino P., Ruocco G., Corcione A. (2021). Multidrug-Resistant Infections and Outcome of Critically Ill Patients with Coronavirus Disease 2019: A Single Center Experience. Microb. Drug Resist..

[43] Garcia-Vidal C., Sanjuan G., Moreno-García E., Puerta-Alcalde P., Garcia-Pouton N., Chumbita M., Fernandez-Pittol M., Pitart C., Inciarte A., Bodro M. (2021). Incidence of co-infections and superinfections in hospitalized patients with COVID-19: A retrospective cohort study. Clin. Microbiol. Infect..

[44] Marcus J.E., Sams V.G., Barsoumian A.E. (2021). Elevated secondary infection rates in patients with coronavirus disease 2019 (COVID-19) requiring extracorporeal membrane oxygenation. Infect. Control Hosp. Epidemiol..

[45] Akagi E.F., Sharma M., Johnson L.B., Szpunar S.M., Riederer K., Saravolatz L.D., Bhargava A. (2021). Clinical features and risk factors for community-onset bloodstream infections among coronavirus disease 2019 (COVID-19) patients. Infect. Control Hosp. Epidemiol..

[46] Wendel Garcia P.D., Fumeaux T., Guerci P., Heuberger D.M., Montomoli J., Roche-Campo F., Schuepbach R.A., Hilty M.P. (2020). Prognostic factors associated with mortality risk and disease progression in 639 critically ill patients with COVID-19 in Europe: Initial report of the international RISC-19-ICU prospective observational cohort. EClinicalMedicine.

[47] Cheng K., He M., Shu Q., Wu M., Chen C., Xue Y. (2020). Analysis of the risk factors for nosocomial bacterial infection in patients with COVID-19 in a tertiary hospital. Risk Manag. Healthc. Policy.

[48] Martinez-Guerra B.A., Gonzalez-Lara M.F., de-Leon-Cividanes N.A., Tamez-Torres K.M., Roman-Montes C.M., Rajme-Lopez S., Villalobos-Zapata G.I., Lopez-Garcia N.I., Martínez-Gamboa A., Sifuentes-Osornio J. (2021). Antimicrobial resistance patterns and antibiotic use during hospital conversion in the COVID-19 pandemic. Antibiotics.

[49] Barry M., Althabit N., Akkielah L., AlMohaya A.E., Alotaibi M., Alhasani S., Aldrees A., AlRajhi A., AlHiji A., Almajid F. (2021). Clinical characteristics and outcomes of hospitalized COVID-19 patients in a MERS-CoV referral hospital during the peak of the pandemic. Int. J. Infect. Dis..

[50] Giacobbe D.R., Battaglini D., Ball L., Brunetti I., Bruzzone B., Codda G., Crea F., De Maria A., Dentone C., Di Biagio A. (2020). Bloodstream infections in critically ill patients with COVID-19. Eur. J. Clin. Investig..

[51] Zhang H., Zhang Y., Wu J., Li Y., Zhou X., Li X., Chen H., Guo M., Chen S., Sun F. (2020). Risks and features of secondary infections in severe and critical ill COVID-19 patients. Emerg. Microbes Infect..

[52] Ripa M., Galli L., Poli A., Oltolini C., Spagnuolo V., Mastrangelo A., Muccini C., Monti G., De Luca G., Landoni G. (2021). Secondary infections in patients hospitalized with COVID-19: Incidence and predictive factors. Clin. Microbiol. Infect..

[53] Contou D., Claudinon A., Pajot O., Micaëlo M., Longuet Flandre P., Dubert M., Cally R., Logre E., Fraissé M., Mentec H. (2020). Bacterial and viral co-infections in patients with severe SARS-CoV-2 pneumonia admitted to a French ICU. Ann. Intensive Care.

[54] Nori P., Cowman K., Chen V., Bartash R., Szymczak W., Madaline T., Punjabi Katiyar C., Jain R., Aldrich M., Weston G. (2021). Bacterial and fungal coinfections in COVID-19 patients hospitalized during the New York City pandemic surge. Infect. Control Hosp. Epidemiol..

[55] Grasselli G., Scaravilli V., Mangioni D., Scudeller L., Alagna L., Bartoletti M., Bellani G., Biagioni E., Bonfanti P., Bottino N. (2021). Hospital-acquired infections in critically-ill COVID-19 patients. Chest.

[56] Cui J., Li M., Cui J., Wang J., Qiang X., Liang Z. (2021). The proportion, species distribution and dynamic trends of bloodstream infection cases in a tertiary hospital in China, 2010–2019. Infection.

[57] (2014). The ProCESS Investigators A Randomized Trial of Protocol-Based Care for Early Septic Shock. N. Engl. J. Med..

[58] Vincent J.L., Sakr Y., Singer M., Martin-Loeches I., MacHado F.R., Marshall J.C., Finfer S., Pelosi P., Brazzi L., Aditianingsih D. (2020). Prevalence and Outcomes of Infection among Patients in Intensive Care Units in 2017. JAMA.

[59] Wicky P.H., Niedermann M.S., Timsit J.F. (2021). Ventilator-associated pneumonia in the era of COVID-19 pandemic: How common and what is the impact?. Crit. Care.

[60] Langford B.J., So M., Raybardhan S., Leung V., Westwood D., MacFadden D.R., Soucy J.P.R., Daneman N. (2020). Bacterial co-infection and secondary infection in patients with COVID-19: A living rapid review and meta-analysis. Clin. Microbiol. Infect..

