# Supplementary material for: Bloodstream Infections in Hospitalized Patients with COVID-19: A Systematic Review and Meta-Analysis
Source: Microorganisms. 2021 Sep 23;9(10):2016. doi: 10.3390/microorganisms9102016 (PMC8540195; doi:10.3390/microorganisms9102016)
Supplement: Supplementary file 1 [file microorganisms-09-02016-s001.zip › PDF S1.pdf]

Bloodstream infections in hospitalized patients with COVID-19: a systematic review and meta-analysis

Mariachiara Ippolito[a], Barbara Simone[a], Carlotta Filisina[a], Francesca Romana Catalanotto[a], Giulia Catalisano[a], Claudia Marino[a], Giovanni Misseri[b], Antonino Giarratano[a,c], Andrea Cortegiani[a,c]

a) Department of Surgical, Oncological and Oral Science (Di.Chir.On.S.), University of Palermo, Italy
b) Fondazione Giglio, Cefalù, Italy
c) Department of Anaesthesia, Intensive Care and Emergency, Policlinico Paolo Giaccone, Palermo, Italy

Corresponding author: Andrea Cortegiani, MD, Department of Surgical, Oncological and Oral Science (Di.Chir.On.S.), University of Palermo, Italy. Department of Anaesthesia, Intensive Care and Emergency, Policlinico Paolo Giaccone, Palermo, Italy, Via del Vespro 129, 90127 Palermo, Italy. Email: andrea.cortegiani@unipa.it; Tel.:+390916552730

Content:

Search strategy

PubMed

Search: **(("bloodstream infection"[All Fields]) OR ("blood stream infection") OR ("bsi"[All Fields]) OR ("blood stream infection"[All Fields]) OR ("bloodstream infection") OR ("crbsi") OR ("clabsi") OR ("clabsi"[All Fields]) OR ("central line associated bloodstream infection"[All Fields]) OR ("catheter related bloodstream infection"[All Fields]) OR ("crbsi"[All Fields]) OR (bacteremia) OR (candidaemia) OR (bacteraemia) OR ("bacteremia"[All Fields]) OR ("candidemia"[All Fields]) OR ("bacteraemia"[All Fields]) OR ("candidaemia"[All Fields]) OR (candidemia) OR ("secondary infection"[All Fields]) OR ("co-infection")) AND ((COVID-19) OR (covid) OR (sars-cov-2))**

("bloodstream infection"[All Fields] OR "blood stream infection"[All Fields] OR "bsi"[All Fields] OR "blood stream infection"[All Fields] OR "bloodstream infection"[All Fields] OR "crbsi"[All Fields] OR "clabsi"[All Fields] OR "clabsi"[All Fields] OR "central line associated bloodstream infection"[All Fields] OR "catheter related bloodstream infection"[All Fields] OR "crbsi"[All Fields] OR ("bacteraemia"[All Fields] OR "bacteremia"[MeSH Terms] OR "bacteremia"[All Fields] OR "bacteraemias"[All Fields] OR "bacteremias"[All Fields]) OR ("candidaemias"[All Fields] OR "candidemia"[MeSH Terms] OR "candidemia"[All Fields] OR "candidaemia"[All Fields]) OR ("bacteraemia"[All Fields] OR "bacteremia"[MeSH Terms] OR "bacteremia"[All Fields] OR "bacteraemias"[All Fields] OR "bacteremias"[All Fields]) OR "bacteremia"[All Fields] OR "candidemia"[All Fields] OR "bacteraemia"[All Fields] OR "candidaemia"[All Fields] OR ("candidemia"[MeSH Terms] OR "candidemia"[All Fields] OR

"candidemias"[All Fields]) OR "secondary infection"[All Fields] OR "co-infection"[All Fields]) AND ("covid 19"[All Fields] OR "covid 19"[MeSH Terms] OR "covid 19 vaccines"[All Fields] OR "covid 19 vaccines"[MeSH Terms] OR "covid 19 serotherapy"[All Fields] OR "covid 19 serotherapy"[Supplementary Concept] OR "covid 19 nucleic acid testing"[All Fields] OR "covid 19 nucleic acid testing"[MeSH Terms] OR "covid 19 serological testing"[All Fields] OR "covid 19 serological testing"[MeSH Terms] OR "covid 19 testing"[All Fields] OR "covid 19 testing"[MeSH Terms] OR "sars cov 2"[All Fields] OR "sars cov 2"[MeSH Terms] OR "severe acute respiratory syndrome coronavirus 2"[All Fields] OR "ncov"[All Fields] OR "2019 ncov"[All Fields] OR (("coronavirus"[MeSH Terms] OR "coronavirus"[All Fields] OR "cov"[All Fields]) AND 2019/11/01:3000/12/31[Date - Publication]) OR ("sars cov 2"[MeSH Terms] OR "sars cov 2"[All Fields] OR "covid"[All Fields] OR "covid 19"[MeSH Terms] OR "covid 19"[All Fields]) OR ("sars cov 2"[MeSH Terms] OR "sars cov 2"[All Fields] OR "sars cov 2"[All Fields]))

Translations

bacteremia: "bacteraemia"[All Fields] OR "bacteremia"[MeSH Terms] OR "bacteremia"[All Fields] OR "bacteraemias"[All Fields] OR "bacteremias"[All Fields]

candidaemia: "candidaemias"[All Fields] OR "candidemia"[MeSH Terms] OR "candidemia"[All Fields] OR "candidaemia"[All Fields]

bacteraemia: "bacteraemia"[All Fields] OR "bacteremia"[MeSH Terms] OR "bacteremia"[All Fields] OR "bacteraemias"[All Fields] OR "bacteremias"[All Fields]

candidemia: "candidemia"[MeSH Terms] OR "candidemia"[All Fields] OR "candidemias"[All Fields]

COVID-19: ("COVID-19" OR "COVID-19"[MeSH Terms] OR "COVID-19 Vaccines" OR "COVID-19 Vaccines"[MeSH Terms] OR "COVID-19 serotherapy" OR "COVID-19 serotherapy"[Supplementary Concept] OR "COVID-19 Nucleic Acid Testing" OR "covid-19 nucleic acid testing"[MeSH Terms] OR "COVID-19 Serological Testing" OR "covid-19

serological testing"[MeSH Terms] OR "COVID-19 Testing" OR "covid-19 testing"[MeSH Terms] OR "SARS-CoV-2" OR "sars-cov-2"[MeSH Terms] OR "Severe Acute Respiratory Syndrome Coronavirus 2" OR "NCOV" OR "2019 NCOV" OR (("coronavirus"[MeSH Terms] OR "coronavirus" OR "COV") AND 2019/11/01[PDAT] : 3000/12/31[PDAT]))

covid: "sars-cov-2"[MeSH Terms] OR "sars-cov-2"[All Fields] OR "covid"[All Fields] OR "covid-19"[MeSH Terms] OR "covid-19"[All Fields]

sars-cov-2: "sars-cov-2"[MeSH Terms] OR "sars-cov-2"[All Fields] OR "sars cov 2"[All Fields]

EMBASE

('bloodstream infection'/exp OR 'bloodstream infection' OR 'bsi' OR 'crbsi' OR 'catheter infection'/exp OR 'catheter infection' OR 'blood stream infection'/exp OR 'blood stream infection' OR 'secondary infection'/exp OR 'secondary infection' OR 'central line infection'/exp OR 'central line infection') AND ('coronavirus disease 2019'/exp OR 'coronavirus disease 2019' OR 'covid-19'/exp OR 'covid-19' OR 'severe acute respiratory syndrome coronavirus 2'/exp OR 'severe acute respiratory syndrome coronavirus 2' OR 'sars-cov-2'/exp OR 'sars-cov-2' OR 'covid'/exp OR 'covid') AND ([embase]/lim OR [medline]/lim OR [pubmed-not-medline]/lim)

WoS

(ALL=(bloodstream infection*) OR ALL=(blood stream infection*) OR ALL=(CRBSI OR BSI OR CLABSI) OR ALL=(catheter related infection*) OR ALL=(central line associated infection*)) AND (ALL=(COVID-19) OR ALL=(sars-cov-2) OR ALL=(covid))
