# Supplementary material for: Bloodstream Infections in Hospitalized Patients with COVID-19: A Systematic Review and Meta-Analysis
Source: Microorganisms. 2021 Sep 23;9(10):2016. doi: 10.3390/microorganisms9102016 (PMC8540195; doi:10.3390/microorganisms9102016)
Supplement: Supplementary file 1 [file microorganisms-09-02016-s001.zip › PDF S2.pdf]

Online supplementary material to:

Bloodstream infections in hospitalized patients with COVID-19: a systematic review and meta-analysis

Mariachiara Ippolito[a], Barbara Simone[a], Carlotta Filisina[a], Francesca Romana Catalanotto[a], Giulia Catalisano[a], Claudia Marino[a], Giovanni Misseri[b], Antonino Giarratano[a,c], Andrea Cortegiani[a,c]

a) Department of Surgical, Oncological and Oral Science (Di.Chir.On.S.), University of Palermo, Italy
b) Fondazione Giglio, Cefalù, Italy
c) Department of Anaesthesia, Intensive Care and Emergency, Policlinico Paolo Giaccone, Palermo, Italy

Corresponding author: Andrea Cortegiani, MD, Department of Surgical, Oncological and Oral Science (Di.Chir.On.S.), University of Palermo, Italy. Department of Anaesthesia, Intensive Care and Emergency, Policlinico Paolo Giaccone, Palermo, Italy, Via del Vespro 129, 90127 Palermo, Italy. Email: andrea.cortegiani@unipa.it; Tel.:+390916552730

CONTENT

Figure S1. Forest plot with the result of the subgroup analysis on the occurrence of bloodstream infection in patients with COVID-19 in multicentre studies.

Figure S2. Forest plot with the result of the subgroup analysis on the occurrence of bloodstream infection in patients with COVID-19 in single centre studies.

Figure S3. Forest plot with the result of the post-hoc subgroup analysis considering only data from studies specifically addressing hospital-acquired bloodstream infections.

Table S1. PRISMA Main Checklist and PRISMA Abstract Checklist

Table S2. Characteristics of bloodstream infections and isolates

Table S3. Quality assessment of studies according to MINORS score for non-comparative studies

Table S4. Quality assessment of studies according to MINORS additional score for comparative studies

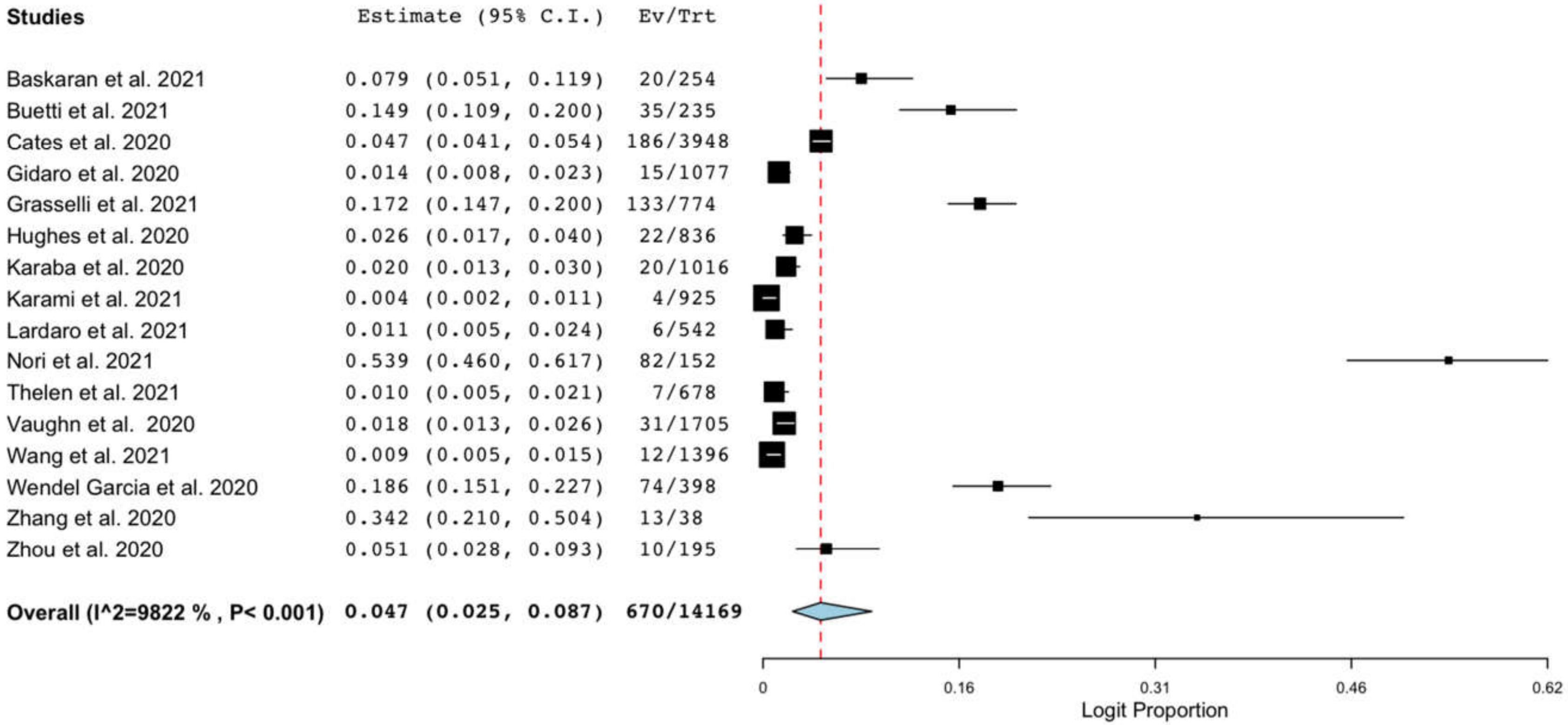

Figure S1. Forest plot with the result of the subgroup analysis on the occurrence of bloodstream infection in patients with COVID-19 in multicentre studies. C.I., confidence interval; Ev, events; Trt, total.

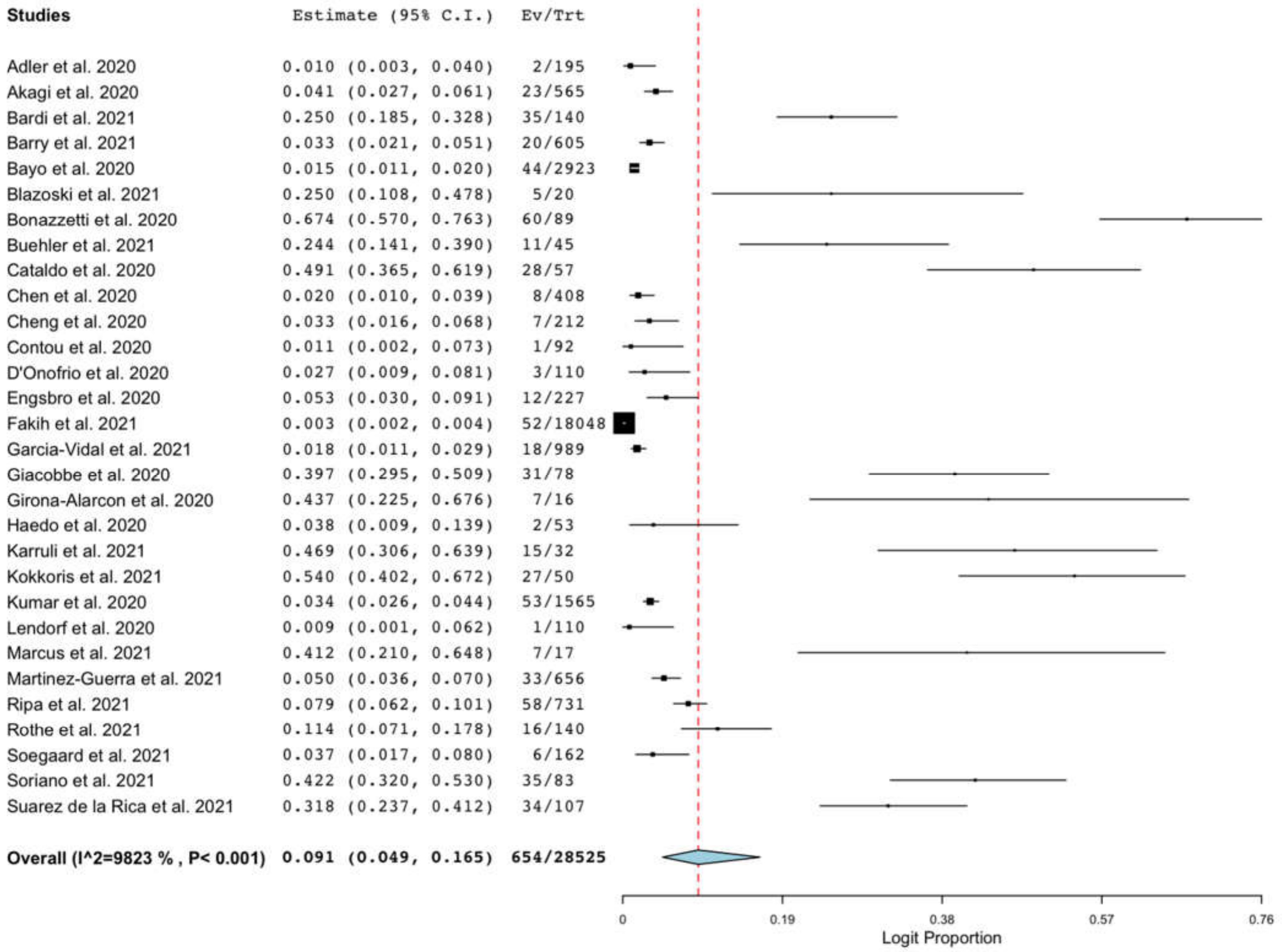

Figure S2. Forest plot with the result of the subgroup analysis on the occurrence of bloodstream infection in patients with COVID-19 in single centre studies. C.I., confidence interval; Ev, events; Trt, total.

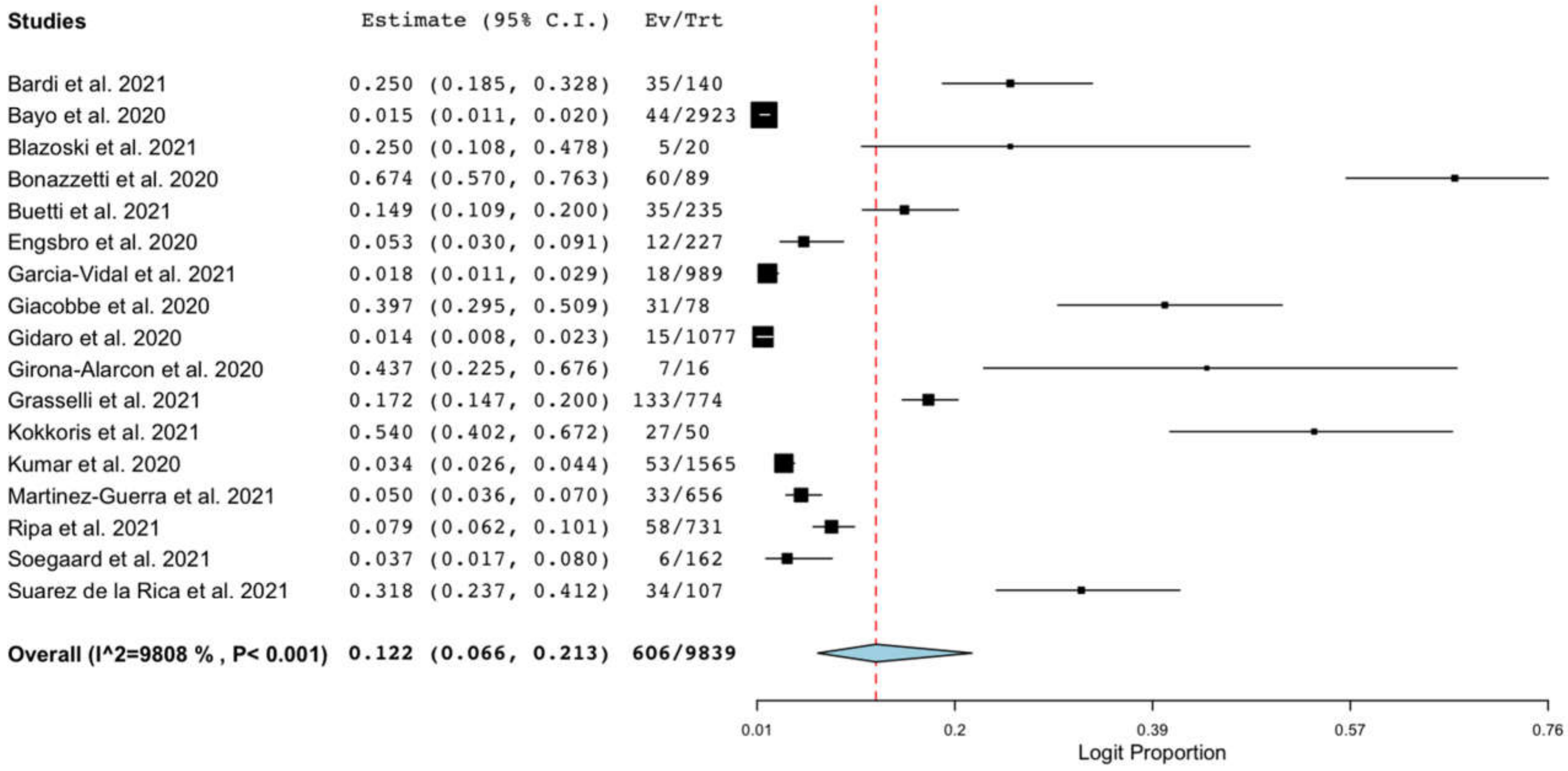
Studies
Estimate (95% C.I.)
Ev/Trt
Bardi et al. 2021 0.250 (0.185, 0.328) 35/140
Bayo et al. 2020 0.015 (0.011, 0.020) 44/2923
Blazoski et al. 2021 0.250 (0.108, 0.478) 5/20
Bonazzetti et al. 2020 0.674 (0.570, 0.763) 60/89
Buetti et al. 2021 0.149 (0.109, 0.200) 35/235
Engsbro et al. 2020 0.053 (0.030, 0.091) 12/227
Garcia-Vidal et al. 2021 0.018 (0.011, 0.029) 18/989
Giacobbe et al. 2020 0.397 (0.295, 0.509) 31/78
Gidaro et al. 2020 0.014 (0.008, 0.023) 15/1077
Girona-Alarcon et al. 2020 0.437 (0.225, 0.676) 7/16
Grasselli et al. 2021 0.172 (0.147, 0.200) 133/774
Kokkoris et al. 2021 0.540 (0.402, 0.672) 27/50
Kumar et al. 2020 0.034 (0.026, 0.044) 53/1565
Martinez-Guerra et al. 2021 0.050 (0.036, 0.070) 33/656
Ripa et al. 2021 0.079 (0.062, 0.101) 58/731
Soegaard et al. 2021 0.037 (0.017, 0.080) 6/162
Suarez de la Rica et al. 2021 0.318 (0.237, 0.412) 34/107
Overall (I^2=9808 % , P< 0.001) 0.122 (0.066, 0.213) 606/9839
0.01
0.2
0.39
0.57
0.76
Logit Proportion

Figure S3. Forest plot with the result of the post-hoc subgroup analysis considering only data from studies specifically addressing hospital-acquired bloodstream infections. C.I., confidence interval; Ev, events; Trt, total.

Section and Topic	Item #	Checklist item	Location where item is reported
**TITLE**			
Title	1	Identify the report as a systematic review.	Page 1
**ABSTRACT**			
Abstract	2	See the PRISMA 2020 for Abstracts checklist.	Page 1
**INTRODUCTION**			
Rationale	3	Describe the rationale for the review in the context of existing knowledge.	Page 1
Objectives	4	Provide an explicit statement of the objective(s) or question(s) the review addresses.	Page 1
**METHODS**			
Eligibility criteria	5	Specify the inclusion and exclusion criteria for the review and how studies were grouped for the syntheses.	Page 2
Information sources	6	Specify all databases, registers, websites, organisations, reference lists and other sources searched or consulted to identify studies. Specify the date when each source was last searched or consulted.	Page 2
Search strategy	7	Present the full search strategies for all databases, registers and websites, including any filters and limits used.	Suppl. Material 1
Selection process	8	Specify the methods used to decide whether a study met the inclusion criteria of the review, including how many reviewers screened each record and each report retrieved, whether they worked independently, and if applicable, details of automation tools used in the process.	Page 2
Data collection process	9	Specify the methods used to collect data from reports, including how many reviewers collected data from each report, whether they worked independently, any processes for obtaining or confirming data from study investigators, and if applicable, details of automation tools used in the process.	Page 2
Data items	10a	List and define all outcomes for which data were sought. Specify whether all results that were compatible with each outcome domain in each study were sought (e.g. for all measures, time points, analyses), and if not, the methods used to decide which results to collect.	Page 2
	10b	List and define all other variables for which data were sought (e.g. participant and intervention characteristics, funding sources). Describe any	Page 2

Section and Topic	Item #	Checklist item	Location where item is reported
		assumptions made about any missing or unclear information.	
Study risk of bias assessment	11	Specify the methods used to assess risk of bias in the included studies, including details of the tool(s) used, how many reviewers assessed each study and whether they worked independently, and if applicable, details of automation tools used in the process.	Page 2
Effect measures	12	Specify for each outcome the effect measure(s) (e.g. risk ratio, mean difference) used in the synthesis or presentation of results.	Page 2
Synthesis methods	13a	Describe the processes used to decide which studies were eligible for each synthesis (e.g. tabulating the study intervention characteristics and comparing against the planned groups for each synthesis (item #5)).	Page 2
	13b	Describe any methods required to prepare the data for presentation or synthesis, such as handling of missing summary statistics, or data conversions.	Page 2
	13c	Describe any methods used to tabulate or visually display results of individual studies and syntheses.	Page 2
	13d	Describe any methods used to synthesize results and provide a rationale for the choice(s). If meta-analysis was performed, describe the model(s), method(s) to identify the presence and extent of statistical heterogeneity, and software package(s) used.	Page 2-3
	13e	Describe any methods used to explore possible causes of heterogeneity among study results (e.g. subgroup analysis, meta-regression).	Page 2-3
	13f	Describe any sensitivity analyses conducted to assess robustness of the synthesized results.	Page 2-3
Reporting bias assessment	14	Describe any methods used to assess risk of bias due to missing results in a synthesis (arising from reporting biases).	NA
Certainty assessment	15	Describe any methods used to assess certainty (or confidence) in the body of evidence for an outcome.	NA
**RESULTS**			
Study selection	16a	Describe the results of the search and selection process, from the number of records identified in the search to the number of studies included in the review, ideally using a flow diagram.	Pages 3 to 13
	16b	Cite studies that might appear to meet the inclusion criteria, but which were excluded, and explain why they were excluded.	Suppl. Mat. 3
Study characteristics	17	Cite each included study and present its characteristics.	Table 1
Risk of bias in studies	18	Present assessments of risk of bias for each included study.	Suppl. Mat. 2
Results of individual studies	19	For all outcomes, present, for each study: (a) summary statistics for each group (where appropriate) and (b) an effect estimate and its precision (e.g. confidence/credible interval), ideally using structured tables or plots.	Figures
Results of syntheses	20a	For each synthesis, briefly summarise the characteristics and risk of bias among contributing studies.	Pages 3 to 13
	20b	Present results of all statistical syntheses conducted. If meta-analysis was done, present for each the summary estimate and its precision (e.g. confidence/credible interval) and measures of statistical heterogeneity. If comparing groups, describe the direction of the effect.	Pages 3 to 13
	20c	Present results of all investigations of possible causes of heterogeneity among study results.	Pages 3 to

Section and Topic	Item #	Checklist item	Location where item is reported
			13
	20d	Present results of all sensitivity analyses conducted to assess the robustness of the synthesized results.	Pages 3 to 13
Reporting biases	21	Present assessments of risk of bias due to missing results (arising from reporting biases) for each synthesis assessed.	NA
Certainty of evidence	22	Present assessments of certainty (or confidence) in the body of evidence for each outcome assessed.	NA
**DISCUSSION**			
Discussion	23a	Provide a general interpretation of the results in the context of other evidence.	Pages 13-14
	23b	Discuss any limitations of the evidence included in the review.	Pages 13-14
	23c	Discuss any limitations of the review processes used.	Pages 13-14
	23d	Discuss implications of the results for practice, policy, and future research.	Pages 13-14
**OTHER INFORMATION**			
Registration and protocol	24a	Provide registration information for the review, including register name and registration number, or state that the review was not registered.	Page 2
	24b	Indicate where the review protocol can be accessed, or state that a protocol was not prepared.	Page 2
	24c	Describe and explain any amendments to information provided at registration or in the protocol.	NA
Support	25	Describe sources of financial or non-financial support for the review, and the role of the funders or sponsors in the review.	Page 14
Competing interests	26	Declare any competing interests of review authors.	Page 14
Availability of data, code and other materials	27	Report which of the following are publicly available and where they can be found: template data collection forms; data extracted from included studies; data used for all analyses; analytic code; any other materials used in the review.	Page 14

Section and Topic	Item #	Checklist item	Reported (Yes/No)
**TITLE**			
Title	1	Identify the report as a systematic review.	Yes
**BACKGROUND**			
Objectives	2	Provide an explicit statement of the main objective(s) or question(s) the review addresses.	Yes
**METHODS**			
Eligibility criteria	3	Specify the inclusion and exclusion criteria for the review.	Yes

Section and Topic	Item #	Checklist item	Location where item is reported
Information sources	4	Specify the information sources (e.g. databases, registers) used to identify studies and the date when each was last searched.	Yes
Risk of bias	5	Specify the methods used to assess risk of bias in the included studies.	No
Synthesis of results	6	Specify the methods used to present and synthesise results.	Yes
**RESULTS**			
Included studies	7	Give the total number of included studies and participants and summarise relevant characteristics of studies.	Yes
Synthesis of results	8	Present results for main outcomes, preferably indicating the number of included studies and participants for each. If meta-analysis was done, report the summary estimate and confidence/credible interval. If comparing groups, indicate the direction of the effect (i.e. which group is favoured).	Yes
**DISCUSSION**			
Limitations of evidence	9	Provide a brief summary of the limitations of the evidence included in the review (e.g. study risk of bias, inconsistency and imprecision).	No
Interpretation	10	Provide a general interpretation of the results and important implications.	Yes
**OTHER**			
Funding	11	Specify the primary source of funding for the review.	No
Registration	12	Provide the register name and registration number.	No

Table S1. PRISMA Main Checklist and PRISMA Abstract Checklist

Authors	Sampling rate [a]	Etiology [b]	MDR [c]	Source [d]
**Adler et al. (2020)**	128/195	*S. pneumoniae 2*	NA	NA
**Akagi et al. (2021)**	266/565	*S. aureus 34.7%*<br>*Escherichia coli 13%*<br>*S. pneumoniae 8.7%*<br>*Enterococcus spp 8.7%*<br>*Candida* spp. *4.3%*	NA	Lung 34.8%<br>Soft-tissue infections 17.4%
**Bardi et al. (2021)**	NA	*Enterococcus spp. 24*<br>*Coagulase-negative staphylococci 16*<br>*P. aeruginosa 2*<br>*S. aureus 2*<br>C. albicans *4*	MRSA 2	19 primary BSI and 16 CRBSI
**Barry et al. (2021)**	332/605	NA	NA	NA
**Baskaran et al. (2021)**	220/254	*Coagulase negative Staphylococcus 13*<br>*Enterococcus spp. 8*<br>*Klebsiella spp. 3*<br>*C. koseri 3*<br>*C. parapsilosis 1*<br>*E. coli 1*<br>*Pseudomonas spp. 1*<br>*S. aureus 1*<br>*H. influenzae 1*	NA	NA
**Bayo et al. (2020)**	NA	*S. epidermidis 38.7%*<br>*E. faecalis 11.4%*<br>*S. aureus 11.4%*<br>*P. aeruginosa 9.1%*<br>*C. albicans 9.1%*	NA	Catheter related 45.5%<br>Unknown 27.3%<br>Respiratory 15.9%<br>Urinary 4.5%
**Blazoski et al. (2021)**	NA	NA	NA	NA
**Bonazzetti et al. (2020)**	NA	*Enterococcus* spp. 53<br>Coagulase-negative	Gram positive MDR 11.7%	Central line-associated 28<br>Pulmonary (VAP) 13

		staphylococci 24<br>*S. aureus 9*<br>Enterobacterales *19*<br>*Candida spp. 3%*	Gram negative MDR 75.8%	Unknown 52
**Buehler et al. (2021)**	NA	*Citrobacter spp.2*<br>*Enterococcus spp. 7*<br>*K. aerogenes 1*<br>*K. Pneumoniae 1*<br>*Moraxella spp. 1*<br>*Candida glabrata 1*<br>*P. aeruginosa 1*	NA	NA
**Buetti et al. (2021)**	NA	*Coagulase-negative Staphylococci 14*<br>*S. aureus 3*<br>*Enterococcus spp. 4*<br>*Enterobacterales 5*<br>*P. aeruginosa 5*<br>*C. albicans 4*		Intra-abdominal 1<br>Skin/soft tissue 2<br>CRBSI 8<br>Pulmonary 8<br>Urinary tract 1<br>Unknown 18
**Cataldo et al. (2020)**	NA	*P. aeruginosa 7*<br>*E. faecalis 3*<br>*E. faecium 6*<br>*K. pneumoniae 1*<br>*E. coli 1*<br>*Candida spp. 6*	VRE 2<br>ESBL 2	NA
**Chen et al. (2020)**	239/408	*Stenotrophomonas maltophilia 3*<br>*Enterococcus faecium 2*<br>*Enterococcus faecalis 1*<br>*Pediococcus lactis 1*<br>*Bacteroides ovatus 1*<br>*Candida parapsilosis 1*	NA	NA
**Contou et al. (2020)**	80/92	*S. aureus* 1	NA	NA
**D'Onofrio et al. (2020)**	110/110	*S. hominis 1*<br>*C. aurimucosum 1*	NA	Pulmonary 1<br>No focus 2

		S. pyogenes 1		
**Engsbro et al. (2020)**	190/227	*Coagulase-negative Staphylococci 4*<br>*S. aureus 2*<br>*Enterococcus faecium 6*<br>*Enterobacter spp. 1*<br>*Klebsiella spp. 1*<br>*E. coli 1*<br>*C. albicans 2*<br>*C. glabrata 1*<br>*C. parapsilosis 1*	NA	Intravascular catheter related 12<br>Pulmonary infection or catheter 3<br>Unknown 2
**Garcia-Vidal et al. (2021)**	267/989	*Coagulase-negative staphylococci* 7<br>*P. aeruginosa* 3<br>*E. faecium* 3<br>*E. coli* 2<br>*S. anginosus* 1<br>*C. albicans* 2		NA

**Giacobbe et al. (2020)**	78/78	*E. aerogenes* 4<br>*E. coli* 1<br>Coagulase-negative staphylococci 11<br>*S. aureus* 6<br>Viridans group streptococci 3<br>*Enterococcus* spp. 12<br>*P. aeruginosa* 2<br>*P. mirabilis* 1<br>*Candida* spp. 3<br>*Prevotella* spp. 1		Lower respiratory tract 10/45<br>Urinary tract 2/45<br>Catheter-related 4/45<br>Unknown 29/45 (episodes)
**Gidaro et al. (2020)**	NA	Gram + 11<br>Gram - 3<br>Fungi 1	NA	Catheter-related 15
**Girona-Alarcon et al. (2020)**	NA	*S. epidermidis 5*<br>*K. pneumoniae 1*<br>*C. albicans 1*	NA	Central line associated 7
**Grasselli et al. (2021)**	NA	*S. aureus 37*<br>*Enterococcus spp. 63*<br>*Coagulase- negative staphylococci 30*<br>*S. pneumoniae 1*<br>*P. aeruginosa 23*<br>*Enterobacterales 43*<br>*Klebsiella spp. 16*<br>*E. coli 10*	NA	Catheter-related 28%

		A. baumannii 15		
**Haedo et al. (2020)**	53/53	NA	NA	NA
**Hughes et al. (2020)**	643/836 (77%)	Enterobacterales 8<br>*S. aureus* 1<br>*P. aeruginosa* 1<br>*C. albicans* 3<br>(isolates)		Central line- associated 3/22,<br>Respiratory 2/22,<br>Urinary tract infections 6/22,<br>Skin and soft tissue infections 3/22,<br>Pelvic inflammatory disease 1/22,<br>Post-partum infection 1/22,<br>Gastrointestinal translocation 1/22,<br>Upper respiratory tract infection 1/22
**Karaba et al. (2020)**	637/1016	*S. aureus 6*<br>*Coagulase-negative Staphylococci 5*<br>*Enterococcus spp.1*<br>*E. coli 5*<br>*K. pneumoniae 2*	MRSA 4	Urinary tract 6<br>Catheter-related 1<br>Gastrointestinal 1<br>Respiratory 1<br>Skin and soft tissue infection 2<br>Unknown 9<br>Other 1
**Karami et al. (2021)**	711/925	*S. pneumoniae 1*<br>*S. aureus 1*<br>*E. coli 2*	NA	Pneumonia 1<br>Cellulitis 1<br>Urinary tract 1<br>Primary bacteremia 1
**Karruli et al. (2021)**	NA	*NA*	Infections reported only if caused by MDR	NA
**Kokkoris et al. (2021)**	NA	*A. Baumannii 7*<br>*K. pneumoniae 4*<br>*Enterococcus spp. 6*	XDR *A. baumannii* 4<br>PDR *A. baumannii* 3<br>*K. pneumoniae*	NA

		C. albicans 4<br>*C. parapsilosis 3*	carbapenemase-positive 4	
**Kumar et al. (2020)**	NA	*S. aureus 10*<br>*S. hominis 1*<br>*S. epidermidis 4*<br>*Group D Streptococcus 5*<br>*E. coli 5*<br>*Klebsiella spp. 2*<br>*Pseudomonas spp. 7*<br>*Serratia spp. 2*<br>*Enterobacter spp. 3*<br>*Proteus spp. 2*<br>*Stenotrophomonas spp. 1*<br>*Candida spp. 11*	MRSA 5	NA
**Lardaro et al. (2021)**	395/542	*S. epidermidis 2*<br>*E. faecalis 2*<br>*Aerococcus spp. 1*<br>*Globicatella 1*<br>*C. striatum 1*<br>*S. pettenkoferi 1*<br>*Acinetobacter 1*	Methicillin-resistance *S. epidermidis* 1	NA
**Lendorf (2020)**	NA	*C. albicans*	NA	NA
**Martinez-Guerra et al. (2021)**	NA	*Coagulase negative staphylococci 14*<br>*Enterobacter complex 7*<br>*Enterococcus spp 6*<br>*P. aeruginosa 3*<br>Other *5*	AmpC producers) 5<br>ESBL producers 1<br>MDR *P. Aeruginosa* 1<br>Azole resistant *Candida* 5	NA
**Nori et al. (2021)**	152/152	*S. aureus* 30%,<br>*S. epidermidis* 12%,<br>*Streptococcus spp.* 10%,	Gram-negative MDR 7, of which CRE 4	Catheter related 23%; respiratory 13%; genitourinary 9%; gastrointestinal 6%;

		Enterococcus spp. 7%,<br>*E. coli* 7%,<br>*P. aeruginosa* 6%,<br>*Candida spp.* 5%,<br>*Klebsiella spp.* 3%,<br>Enterobacter spp. 3%<br>*Candida* spp. 5%		multiple 30%<br>oropharyngeal 2%<br>skin 2%<br>other 2 %<br>unknown 6%
**Ripa et al. (2021)**	NA	*E. coli* 5;<br>*K. pneumoniae* 1;<br>*S. aureus* 2;<br>*E. faecalis* 6;<br>*E. faecium* 3;<br>*A. baumannii* 5;<br>*P. aeruginosa* 3;<br>*C. albicans* 5<br>coagulase-negative staphylococci 53	MRSA 1;<br>VRE 4;<br>3°-gen cephalosporin-resistant E. cloacae 2;<br>3°-gen cephalosporin-resistant E. coli 4;<br>carbapenem-resistant *A. baumannii* 7;<br>carbapenem-resistant *P. aeruginosa* 2	NA
**Rothe et al. (2021)**	118/140<br>(57 follow-up)	*E. coli 2,*<br>*S. aureus 1,*<br>*S. epidermidis 2*<br>*K. oxytoca 1,*<br>*K. pneumoniae 1*<br>*P. aeruginosa 1*<br>*S. epidermidis 4*<br>*C. albicans 2,*<br>*Enterococcus spp. 3*<br>*K. varicola 1*	VRE 2	NA
**Søgaard et al. (2021)**	127/162	*S. epidermidis 1*<br>*E. coli 1*<br>*C. koseri 1*<br>*P. aeruginosa 1*<br>*S. pneumoniae 1*<br>*C. albicans 1*	No MDR among BSI	Catheter-associated 2<br>Pulmonary 1<br>Urinary tract 1<br>Unknown 1<br>Primary CA BSI 1
**Soriano et al. (2021)**	NA	NA	NA	Primary 28

				Catheter-related 7
**Suarez de la Rica et al. (2021)**	NA	*Enterococcus spp. 19*<br>*Klebsiella spp. 5*<br>*P. aeruginosa 2*<br>*Candida spp. 12*<br>*S. aureus 6*<br>*E. coli 4*	NA	NA
**Thelen et al. (2021)**	678/678	*E. coli 2*<br>*K. pneumoniae 1*<br>*P. aeruginosa 1*<br>*S. pneumoniae 2*<br>*S. aureus 1*	NA	NA
**Vaughn et al. (2020)**	1063/1705	NA	NA	NA
**Wang et al. (2021)**	969/1396	*E. coli 2*<br>*K. pneumoniae 1*<br>*K. variicola 1*<br>*Proteus mirabilis 4 S. aureus 2*<br>*S. epidermidis 1*	MRSA 1	Urinary tract 6<br>Central venous access related 1<br>Unclear 5
**Zhang et al. (2020)**	NA	*S. haemolyticus* 2<br>*C. albicans* 4<br>*Cryptococcus spp.* 1<br>*T. asahii* 1<br>*E. coli* 2<br>*E. faecium* 1<br>*K. pneumoniae* 3		NA

Table S2. Characteristics of bloodstream infections and isolates
The table shows the main characteristics of the bloodstream infections and microbiological isolates.
[a] The column “sampling rate” shows the proportion of patients tested with a blood culture on the total number of included patients, as reported by the authors, when available.
[b] The column “etiology” shows the microbiological characteristics of the isolates, as reported by the authors, when available.

[c] The column "MDR" shows the number or percentage and the types of multidrug resistant microorganisms grown in the cultures, when available.
[d] The column "source" shows the diagnosed or suspected source of the bloodstream infection, presented as number of cases when available, or percentages.

BSI, bloodstream infection; CRBI, catheter-related bloodstream infection; CRE, Carbapenem-Resistant Enterobacteriaceae; ESBL, extended-spectrum beta-lactamase; ICU, intensive care unit; MDR, Multi-Drug Resistance; MRSA, Methicillin-Resistant Staphylococcus Aureus; NA, not available; PDR, pandrug-resistant ; VAP, Ventilator-associated pneumonia; VRE, Vancomycin-Resistant Enterococcus; XDR, extensively drug-resistant.

Authors (year)	Aim of the study	Inclusion of consecutive patients	Prospective collection of data	Endpoint appropriate to the study aim	Unbiased evaluation of endpoints	Follow up period appropriate to the major endpoint	Loss to follow up not exceeding 5%	Sample calculation	Score
**Adler et al. (2020)**	2 Reported and adequate	2 Reported and adequate	1* No protocol registration	2 Reported and adequate	2 Reported and adequate	2 Reported and adequate	2 Reported and adequate	0 Not reported	13/10
**Akagi et al. (2021)**	2 Reported and adequate	2 Reported and adequate	1* No protocol registration	2 Reported and adequate	2 Reported and adequate	2 Reported and adequate	2 Reported and adequate	0 Not reported	13/18
**Bardi et al. (2021)**	2 Reported and adequate	2 Reported and adequate	1* No protocol registration	2 Reported and adequate	2 Reported and adequate	2 Reported and adequate	2 Reported and adequate	0 Not reported	13/16
**Barry et al. (2021)**	2 Reported and adequate	2 Reported and adequate	1* No protocol registration	2 Reported and adequate	2 Reported and adequate	2 Reported and adequate	2 Reported and adequate	0 Not reported	13/16
**Baskaran et al. (2021)**	2 Reported and	2 Reported and adequate	1* No protocol registration	2 Reported and	2 Reported and	2 Reported and adequate	2 Reported and	0 Not reported	13/16

	adequate			adequate	adequate		adequate		
**Bayo et al. (2020)**	2 Reported and adequate	1* Participating sites entered data for either: all identified patients, or a random selection of at least ten patients from across their eligible cohort. Random sampling wasn't standardized	1* No protocol registration	2 Reported and adequate	2 Reported and adequate	2 Reported and adequate	2 Reported and adequate	0 Not reported	12/16
**Blazoski et al. (2021)**	2 Reported and adequate	2 Reported and adequate	1* No protocol registration	2 Reported and adequate	2 Reported and adequate	2 Reported and adequate	2 Reported and adequate	0 Not reported	13/16
**Bonazzetti et al. (2020)**	2 Reported and adequate	2 Reported and adequate	1* No protocol registration	2 Reported and adequate	2 Reported and adequate	2 Reported and adequate	2 Reported and adequate	0 Not reported	13/10
**Buehler et al. (2021)**	2 Reported and adequate	2 Reported and adequate	1* No protocol registration	2 Reported and adequate	2 Reported and adequate	2 Reported and adequate	2 Reported and adequate	0 Not reported	13/16
**Buetti et al. (2021)**	2 Reported and adequate	2 Reported and adequate	1* No protocol registration	2 Reported and adequate	2 Reported and adequate	2 Reported and adequate	2 Reported and adequate	0 Not reported	13/16

**Cataldo et al. (2020)**	2 Reported and adequate	2 Reported and adequate	1* No protocol registration	2 Reported and adequate	2 Reported and adequate	2 Reported and adequate	2 Reported and adequate	0 Not reported	13/16
**Cates et al. (2020)**	2 Reported and adequate	2 Reported and adequate	1* No protocol registration	2 Reported and adequate	2 Reported and adequate	2 Reported and adequate	2 Reported and adequate	0 Not reported	13/16
**Chen et al. (2020)**	2 Reported and adequate	2 Reported and adequate	1* No protocol registration	2 Reported and adequate	2 Reported and adequate	2 Reported and adequate	2 Reported and adequate	0 Not reported	13/16
**Cheng et al. (2020)**	2 Reported and adequate	2 Reported and adequate	1* No protocol registration	2 Reported and adequate	2 Reported and adequate	2 Reported and adequate	2 Reported and adequate	0 Not reported	13/16
**Contou et al. (2020)**	2 Reported and adequate	2 Reported and adequate	1* No protocol registration	2 Reported and adequate	2 Reported and adequate	2 Reported and adequate	2 Reported and adequate	0 Not reported	13/16
**D'Onofrio et al. (2020)**	2 Reported and adequate	2 Reported and adequate	1* Secondary analysis of part of a registered study	2 Reported and adequate	2 Reported and adequate	1* The follow up does not include the whole hospitalization period.	2 Reported and adequate	0 Not reported	12/16
**Engsbro et al. (2020)**	2 Reported and adequate	2 Reported and adequate	1* No protocol registration	2 Reported and adequate	2 Reported and adequate	2 Reported and adequate	2 Reported and adequate	0 Not reported	13/16
**Fakih et al. (2021)**	2 Reported and adequate	2 Reported and adequate	1* No protocol registration	2 Reported and adequate	2 Reported and adequate	2 Reported and adequate	2 Reported and adequate	0 Not reported	13/16
**Garcia-Vidal et al. (2021)**	2 Reported and	2 Reported and adequate	1* No protocol registration	2 Reported and	2 Reported and	2 Reported and adequate	2 Reported and	0 Not reported	13/16

	adequate			adequate	adequate		adequate		
**Giacobbe et al. (2020)**	2 Reported and adequate	2 Reported and adequate	1* No protocol registration	2 Reported and adequate	2 Reported and adequate	2 Reported and adequate	2 Reported and adequate	0 Not reported	13/16
**Gidaro et al. (2020)**	2 Reported and adequate	2 Reported and adequate	1* No protocol registration	2 Reported and adequate	2 Reported and adequate	2 Reported and adequate	2 Reported and adequate	0 Not reported	13/16
**Girona-Alarcon et al. (2020)**	2 Reported and adequate	2 Reported and adequate	1* No protocol registration	2 Reported and adequate	2 Reported and adequate	2 Reported and adequate	2 Reported and adequate	0 Not reported	13/16
**Grasselli et al. (2021)**	2 Reported and adequate	2 Reported and adequate	2 Reported and adequate	2 Reported and adequate	2 Reported and adequate	2 Reported and adequate	2 Reported and adequate	0 Not reported	14/16
**Haedo et al. (2020)**	2 Reported and adequate	2 Reported and adequate	1* No protocol registration	2 Reported and adequate	2 Reported and adequate	2 Reported and adequate	2 Reported and adequate	0 Not reported	13/16
**Hughes et al. (2020)**	2 Reported and adequate	2 Reported and adequate	1* No protocol registration	2 Reported and adequate	2 Reported and adequate	1* Median follow up 6 days	2 Reported and adequate	0 Not reported	12/16
**Karaba et al. (2020)**	2 Reported and adequate	2 Reported and adequate	1* No protocol registration	2 Reported and adequate	2 Reported and adequate	2 Reported and adequate	2 Reported and adequate	0 Not reported	13/16
**Karami et al. (2021)**	2 Reported and adequate	2 Reported and adequate	1* No protocol registration	2 Reported and adequate	2 Reported and adequate	2 Reported and adequate	2 Reported and adequate	0 Not reported	13/16
**Karruli et al. (2021)**	2 Reported	2 Reported and	1* No protocol	2 Reported	2 Reported	2 Reported and	2 Reported	0 Not reported	13/16

	and adequate	adequate	registration	and adequate	and adequate	adequate	and adequate		
**Kokkoris et al. (2021)**	2 Reported and adequate	2 Reported and adequate	1* No protocol registration	2 Reported and adequate	2 Reported and adequate	2 Reported and adequate	2 Reported and adequate	0 Not reported	13/16
**Kumar et al. (2020)**	2 Reported and adequate	2 Reported and adequate	1* No protocol registration	2 Reported and adequate	2 Reported and adequate	2 Reported and adequate	2 Reported and adequate	0 Not reported	13/16
**Lardaro et al. (2021)**	2 Reported and adequate	2 Reported and adequate	1* No protocol registration	2 Reported and adequate	2 Reported and adequate	2 Reported and adequate	2 Reported and adequate	0 Not reported	13/16
**Lendorf (2020)**	2 Reported and adequate	2 Reported and adequate	1* No protocol registration	2 Reported and adequate	2 Reported and adequate	2 Reported and adequate	2 Reported and adequate	0 Not reported	13/16
**Marcus et al. (2021)**	2 Reported and adequate	2 Reported and adequate	1* No protocol registration	2 Reported and adequate	2 Reported and adequate	2 Reported and adequate	2 Reported and adequate	0 Not reported	13/16
**Martinez-Guerra et al. (2021)**	2 Reported and adequate	2 Reported and adequate	1* No protocol registration	2 Reported and adequate	2 Reported and adequate	2 Reported and adequate	1* Loss to follow up > 5%	0 Not reported	12/16
**Nori et al. (2021)**	2 Reported and adequate	2 Reported and adequate	1* No protocol registration	2 Reported and adequate	2 Reported and adequate	2 Reported and adequate	2 Reported and adequate	0 Not reported	13/16
**Ripa et al. (2021)**	2 Reported and adequate	2 Reported and adequate	2 Reported and adequate	2 Reported and adequate	2 Reported and adequate	2 Reported and adequate	2 Reported and adequate	0 Not reported	14/16
**Rothe et al. (2021)**	2 Reported	2 Reported and	1* No protocol	2 Reported	2 Reported	2 Reported and	2 Reported	0 Not reported	13/16

	and adequate	adequate	registration	and adequate	and adequate	adequate	and adequate		
**Søgaard et al. (2021)**	2 Reported and adequate	2 Reported and adequate	1* No protocol registration	2 Reported and adequate	2 Reported and adequate	2 Reported and adequate	2 Reported and adequate	0 Not reported	13/16
**Soriano et al. (2021)**	2 Reported and adequate	2 Reported and adequate	1* No protocol registration	2 Reported and adequate	2 Reported and adequate	2 Reported and adequate	2 Reported and adequate	0 Not reported	13/16
**Suarez de la Rica et al. (2021)**	2 Reported and adequate	2 Reported and adequate	1* No protocol registration	2 Reported and adequate	2 Reported and adequate	2 Reported and adequate	2 Reported and adequate	0 Not reported	13/16
**Thelen et al. (2021)**	2 Reported and adequate	2 Reported and adequate	1* No protocol registration	2 Reported and adequate	2 Reported and adequate	2 Reported and adequate	2 Reported and adequate	0 Not reported	13/16
**Vaughn et al. (2020)**	2 Reported and adequate	1* Hospitals unable to abstract all cases followed a pseudo-randomization procedure (in which daily eligible cases were sorted by time stamp of discharge and included in order of smallest minute value until	1* No protocol registration	2 Reported and adequate	2 Reported and adequate	2 Reported and adequate	2 Reported and adequate	0 Not reported	12/16

		abstraction capacity was reached)							
**Wang et al. (2021)**	2 Reported and adequate	2 Reported and adequate	1* No protocol registration	2 Reported and adequate	2 Reported and adequate	2 Reported and adequate	1* Loss to follow up > 5%	0 Not reported	12/16
**Wendel Garcia et al. (2020)**	2 Reported and adequate	2 Reported and adequate	1* No protocol registration	2 Reported and adequate	2 Reported and adequate	2 Reported and adequate	2 Reported and adequate	0 Not reported	13/16
**Zhang et al. (2020)**	2 Reported and adequate	2 Reported and adequate	1* No protocol registration	2 Reported and adequate	2 Reported and adequate	2 Reported and adequate	2 Reported and adequate	0 Not reported	13/16
**Zhou et al. (2020)**	2 Reported and adequate	2 Reported and adequate	1* No protocol registration	2 Reported and adequate	2 Reported and adequate	2 Reported and adequate	2 Reported and adequate	0 Not reported	13/16

Table S3. Quality assessment of studies according to MINORS score for non-comparative studies.

Qualitative assessment was performed using the MINORS score. The eight items are scored 0 (not reported), 1 (reported but inadequate) or 2 (reported and adequate). The global ideal score being 16 for non-comparative studies.

* For the domains judged as ‘reported but inadequate’, the table provides a brief explanation of the detected reason for inadequacy. Please also see the additional score for comparative studies at Table S3

	An adequate control group	Contemporary groups	Baseline equivalence of groups	Adequate statistical analyses	Additional score for comparative studies
**Bayo et al. (2020)**	2<br>Reported and adequate	1*<br>Historical cohort	1*<br>Potential confounders among baseline characteristics	1*<br>Unadjusted analysis	5/8
**Buetti et al. (2021)**	2<br>Reported and adequate	1*<br>Historical cohort	2<br>Reported and adequate	2<br>Reported and adequate	7/8
**Cataldo et al. (2020)**	2<br>Reported and adequate	1*<br>Historical cohort	1*<br>Potential confounders among baseline characteristics	1*<br>Unadjusted analysis	5/8
**Cates et al. (2020)**	2<br>Reported and adequate	1*<br>Historical cohort	1*<br>Potential confounders among baseline characteristics	2<br>Reported and adequate	6/8
**D'Onofrio et al. (2020)**	2<br>Reported and adequate	1*<br>Historical cohort	1*<br>Potential confounders among baseline characteristics	1*<br>Unadjusted analysis	5/8
**Engsbro et al. (2020)**	2<br>Reported and adequate	2<br>Reported and adequate	1*<br>Potential confounders among baseline characteristics	1*<br>Unadjusted analysis	6/8
**Fakih et al. (2021)**	2<br>Reported and adequate	1*<br>Historical cohort	1*<br>Potential confounders among baseline	1*<br>Unadjusted analysis	5/8

			characteristics		
**Gidaro et al. (2020)**	2<br>Reported and adequate	2<br>Reported and adequate	1*<br>Potential confounders among baseline characteristics	2<br>Reported and adequate	7/8
**Hughes et al. (2020)**	2<br>Reported and adequate	1*<br>Historical cohort	1*<br>Potential confounders among baseline characteristics	1*<br>Unadjusted analysis	5/8
**Marcus et al. (2021)**	2<br>Reported and adequate	1*<br>Historical cohort	1*<br>Potential confounders among baseline characteristics	1*<br>Unadjusted analysis	5/8
**Thelen et al. (2021)**	2<br>Reported and adequate	1*<br>Historical cohort	1*<br>Potential confounders among baseline characteristics	1*<br>Unadjusted analysis	5/8

Table S4. **Quality assessment of studies according to MINORS additional score for comparative studies.**

Qualitative assessment was performed using the MINORS score. The four items are scored 0 (not reported), 1 (reported but inadequate) or 2 (reported and adequate). The global ideal score being 24 (sum of non-comparative and comparative study items) for comparative studies.

* For the domains judged as ‘reported but inadequate’, the table provides a brief explanation of the detected reason for inadequacy.
